# Proposal of Troglocephalinae n. subfam. (Monogenea: Monocotylidae) to accommodate existing and two new monocotylids from the gills of rhinopristiform shovelnose rays

**DOI:** 10.1007/s11230-024-10174-z

**Published:** 2024-07-17

**Authors:** David B. Vaughan, Haakon Hansen, Leslie A. Chisholm

**Affiliations:** 1 Aquatic Animal Health Research, Two Oceans Aquarium, Cape Town, South Africa; 2 Present Address: School of Access Education, Tertiary Education Division, Central Queensland University, Rockhampton, QLD Australia; 3 Present Address: Coastal Marine Ecosystems Research Centre, Central Queensland University, Rockhampton, QLD Australia; 4 Norwegian Veterinary Institute, P. O. Box 64, 1433 Ås, Norway; 5 Parasitology Section, South Australian Museum, North Terrace, Adelaide, SA 5000 Australia; 6 Faculty of Sciences, Engineering and Technology, School of Biological Sciences, University of Adelaide, North Terrace, Adelaide, SA 5005 Australia

## Abstract

Troglocephalinae **n. subfam.** is proposed for *Spinuris* Doran, 1953, *Neoheterocotyle* Hargis, 1955, *Anoplocotyloides* Young, 1967, *Troglocephalus rhinobatidis* Young, 1967 (previously *incertae sedis*), *Nonacotyle pristis* Ogawa, 1991, *Mehracotyle insolita* Neifar, Euzet & Ben Hassine, 2002, *Scuticotyle cairae* **n. gen. et sp.**, and *Brancheocotyle imbricata* **n. gen. et sp.** All members of the proposed new subfamily are gill parasites of shovelnose rays of the order Rhinopristiformes. The subfamilies Heterocotylinae Chisholm, Wheeler & Beverley-Burton, 1995, and Dasybatotreminae Bychowsky, 1957, are amended to exclude *Spinuris*, *Nonacotyle*, *Neoheterocotyle*, and *Anoplocotyloides* and *Mehracotyle*, respectively. Heterocotylinae includes gill parasites of members of the orders Myliobatiformes and Torpediniformes. Dasybatotreminae includes parasites of the gills and pharyngeal cavity of members of the orders Myliobatiformes and Rajiformes. A revised phylogeny of the Monocotylidae Taschenberg, 1879 is presented and discussed, based on 28S rDNA sequences, including new sequences for *Myliocotyle pteromylaei* Neifer, Euzet & Ben Hassine, 1999, *Heterocotyle tokoloshei* Vaughan & Chisholm, 2010, *Neoheterocotyle robii* Vaughan & Chisholm, 2010, and the two newly proposed species and genera. Additional locality records are also provided for Monocotylidae from off South Africa.

**Supplementary Information:**

The online version contains supplementary material available at 10.1007/s11230-024-10174-z.

## Introduction

Monocotylids (Monogenea: Monocotylidae Taschenberg, 1879) are parasites of chondrichthyans of marine, brackish and fresh waters. Their host microhabitats are diverse, including the gill lamellae, pharyngeal cavity, skin surface, nasal tissue, urogenital system, and inner wall of the body cavity (Chisholm and Whittington 1998a; Derouiche et al. 2019, Bullard et al. 2021; Ruiz-Escobar et al. 2022). Traditionally, the morphology of the haptor, including the number of loculi (or a 3-part attachment organ; Bullard et al. 2021), and the presence of a variety of ventral and dorsal haptoral structures, presumably to facilitate attachment to the variety of host microhabitats, has been of primary importance in discriminating higher-level monocotylid taxa (Chisholm and Whittington 1998a; Bullard et al. 2021). The morphology of the male copulatory organ and vagina is useful for discriminating between species. Comparatively little attention has been afforded to the importance of the anterior head region, and its structures that might demonstrate relatedness between taxa. An inconsistent approach exists for including details of the head glands of the anterior head region historically. Some additional details, such as the presence of ventral pits in the anterior head region (e.g., Hargis 1955; Young 1967, for *Neoheterocotyle* Hargis, 1955 species) or differences in the nature of the gland-duct openings, have largely been ignored. Notably, Young (1967) described both *Neoheterocotyle rhinobatidis* (Young, 1967) Chisholm, 1994 and *Troglocephalus rhinobatidis* Young, 1967 in the same work, yet only described ventral pits for the latter. This feature is confirmed in all *Neoheterocotyle* species (Chisholm and Whittington 1997). Young (1967) also referred to the ventral pits as “clear markings of unknown nature” for *Anoplocotyloides papillatus* (Doran, 1953), Young, 1967, perhaps because the function of these ventral pits has never adequately been experimentally demonstrated. Some of these subtle characters are undoubtedly difficult to observe, but with current, modern technology, a renewed focus on this region in monocotylids is warranted. Recently, workers have begun to include the relative importance of these subtle characters in phylogenetic analyses of the family (e.g., Boeger et al. 2014; Bullard et al. 2021).

Monocotylidae currently contains nine subfamilies: Calicotylinae Monticelli, 1903, Cathariotrematinae Bullard in Bullard, Warren & Dutton, 2021, Dasybatotreminae Bychowsky, 1957, Decacotylinae Chisholm, Wheeler & Beverley-Burton, 1995, Euzetiinae Chisholm & Whittington, 2001, Heterocotylinae Chisholm, Wheeler & Beverley-Burton, 1995, Loimoinae Price, 1936, Merizocotylinae Johnston & Tiegs, 1922 and Monocotylinae Taschenberg, 1879. Heterocotylinae was proposed in the morphological revision of the family of Chisholm et al. (1995) and was considered monophyletic based on two purported apomorphies: four dorsal haptoral accessory structures, and their rounded shape. In addition to *Heterocotyle* Scott, 1904 and *Potamotrygonocotyle* Mayes, Brooks & Thorson, 1981, these authors included *Neoheterocotyle*, *Nonacotyle* Ogawa, 1991 and *Spinuris* Doran, 1953 in the subfamily, which have four, six or 14 projecting, mostly spiculate dorsal haptoral sclerites. This variation was considered a modification of the character states within the subfamily (Chisholm et al. 1995). At that time, Heterocotylinae was considered the only subfamily to include representatives with dorsal haptoral accessory structures; however, Decacotylinae, which was also proposed in the same publication, was later revised to include dorsal haptoral accessory structures (Chisholm and Whittington 1998b). Subsequently, *Heliocotyle* Neifar, Euzet & Ben Hassine, 1999, and *Malalophus* Chisholm & Whittington, 2009 were proposed as representatives of Heterocotylinae with only a single dorsal haptoral accessory structure. Euzetiinae includes two genera, *Euzetia* Chisholm & Whittington, 2001, without these structures, and *Denarycotyle* Pulido-Flores, Monks & Violante-González, 2015 with them present, indicating that the presence of dorsal haptoral accessory structures is not restricted to Heterocotylinae, and that their presence or absence is also characteristic within Euzetiinae.

Dasybatotreminae was originally erected by Bychowsky (1957) to accommodate *Dasybatotrema dasybatis* (MacCallum, 1916), Price, 1938 from the gills of the marine rays *Dasyatis pastinaca* (Linnaeus) and *Pastinachus centrourus* (Mitchill) [now *Bathytoshia centroura* (Mitchill)]. The proposal of Dasybatotreminae was based on the presence of numerous anterior gland-duct openings along the anterior edge of the “adoral sucker” (anterior head region), and the morphology of the hamulus consisting of a reduced superficial root and elongated deep root in the type-species. Yamaguti (1963) subsequently assigned *D. dasybatis* to Monocotylinae, thus synonymising Dasybatotreminae with the former subfamily, without explanatory comment. Chisholm et al. (1995) revised the family based on morphology, reinstated Dasybatotreminae, and included the additional genera *Anoplocotyloides*, *Timofeevia*, and *Troglocephalus* based on two apomorphies: the elongated deep root of the hamulus, and numerous anterior gland-duct openings. The inclusion of *Tr. rhinobatidis* in Dasybatotreminae would later prove problematic. In the subsequent molecular phylogenetic analysis of the family by Chisholm et al. (2001a), as the sole representative of Dasybatotreminae, *Tr. rhinobatidis* grouped together with three *Neoheterocotyle* species in a monophyletic group, separate to *Heterocotyle capricornensis* Chisholm & Whittington, 1996. This presented the Heterocotylinae as paraphyletic but also suggested that the presence of ventral pits on the anterior head region of *Tr. rhinobatidis* and *Neoheterocotyle* species was a unifying character. Given the low taxa resolution of this initial molecular phylogeny, the monotypic nature of *Troglocephalus*, and to avoid transferring *Tr. rhinobatidis* to Heterocotylinae, *Tr. rhinobatidis* was rendered *incertae sedis* (Chisholm et al. 2001a). A year later, another *Troglocephalus*-like monocotylid, *Mehracotyle insolita* Neifar, Euzet & Ben Hassine, 2002 was proposed by Neifar et al. (2002). These authors were aware of the problems resulting from profound changes to the classification of Heterocotylinae if *Tr. rhinobatidis* was transferred to this subfamily, and specifically to avoid these problems, they chose to consider *Me. insolita* a member of Dasybatotreminae based on the earlier morphological work by Chisholm et al. (1995). In making their decision regarding *Me. insolita*, they stated that it was provisional, and stopped short of formally reassigning *Troglocephalus* back to Dasybatotreminae. In the same work, Neifar et al. (2002) also indicated that the presence of ventral pits on the anterior head region in *Me. insolita* and *Tr. rhinobatidis* were also shared with *A. papillatus*, and that all three species were from rhinopristiform hosts. In addition, these authors stated that the larvae observed for *Me. insolita* demonstrated a morphological similarity in the permutation of larval ciliated cells with larvae described for *Neoheterocotyle* species, a similar conclusion reached by Chisholm (1998) for larvae of *Ne. rhinobatidis* and *Tr. rhinobatidis*, suggesting a close relationship. This close relationship was also supported by the investigations of spermiogenesis and sperm ultrastructure by Watson (1997) for the latter two species.

Both *Dasybatotrema* species, *D. dasybatis* and *D. spinosum* Timofeeva, 1983 have a distinct arrangement of gland-duct openings of the anterior head region to those of other monocotylids and have no ventral pits. The unique morphology of these gland-duct openings is also shared by *Timofeevia rajae* (Timofeeva, 1983) Chisholm, Wheeler & Beverley-Burton, 1995, which also has no ventral pits. Recently, Dasybatotreminae was amended to accommodate a new genus, *Peruanocotyle* Chero, Cruces, Sáez & Luque, 2018, for *P. chisholmae* Chero, Cruces, Sáez & Luque, 2018. A second species of *Peruanocotyle*, *P. pelagica* Ruiz-Escobar, Torres-Carrera & Ramos-Sánchez, 2022 was subsequently described. Both *Peruanocotyle* species demonstrate the same anterior head region gland-duct opening arrangement as *Dasybatotrema* species and *Ti. rajae*, and the absence of ventral pits. Except for *Ti. rajae* as a parasite of a rajiform skate, these species are all parasites of myliobatiform stingrays. Dasybatotreminae can thus be separated into two groups of taxa based on the morphology of the anterior head region. Similarly, additional taxa from rhinopristiform hosts that share morphological similarities in the anterior head region with *A. papillatus*, *Me. insolita* and *Tr. rhinobatidis*, are currently classified under Heterocotylinae. Of these, only *Neoheterocotyle* species are represented currently in molecular phylogenies, but consistently reflect the original representation of Heterocotylinae as polyphyletic, remaining separate from other heterocotylinid species currently, even as family and subfamily resolution has improved over time (see Fehlauer-Ale and Littlewood 2011; Boeger et al. 2014; Vaughan et al. 2016; Derouiche et al. 2019; Bullard et al. 2021; Chero et al. 2021; Dalrymple et al. 2022; Ruiz-Escobar et al. 2022).

During the parasitological investigation of elasmobranchs off South Africa, two new monocotylid species representing new genera were collected from the shovelnose ray, *Acroteriobatus annulatus* (Smith). These monocotylids share morphological similarities of the anterior head region with *A. papillatus*, *Me. insolita*, *Neoheterocotyle* species, *No. pristis*, *Spinuris* species, and *Tr. rhinobatidis*. Based on the shared morphological features of the anterior head region in these species from rhinopristiform hosts, additional historic evidence in the literature of larval similarity between *Tr. rhinobatidis*, *Neoheterocotyle* species, and *Me. insolita*, similar sperm ultrastructure for *Ne. rhinobatidis* and *Tr. rhinobatidis*, and historic and new molecular phylogenetic information, a new subfamily is proposed herein, requiring the re-evaluation of Heterocotylinae and Dasybatotreminae. The two new species are described, and additional data are presented for other monocotylids collected during the same period, from South Africa.

## Materials and methods

In April 2008, during the South African government department of Fisheries and Environment’s commercial demersal sole fishery survey, two female and one male *Dasyatis chrysonota* (Smith), and one male *Galeorhinus galeus* (Linnaeus) were collected as dead trawl bycatch on board the fisheries research vessel Africana off South Africa’s South coast (off Cape Agulhas). The gill arches were dissected, and nasal fossae inspected on board the Africana. Specimens of *Heterocotyle pastinacae* Scott, 1904 were recovered from the gills of *D. chrysonota*, and *Cathariotrema selachii* (MacCallum, 1916) from the nasal fossae of *G. galeus*. In February 2010, six *Ac. annulatus*, and a single, large female *Aetomylaeus bovinus* (Geoffroy Saint-Hilaire) were obtained live from the traditional commercial seine-net fishermen operating off Muizenberg beach (34°06’13” S, 18°29’00” E) in False Bay, South Africa, collected by staff of Two Oceans Aquarium, Cape Town. The *Ae. bovinus* and one *Ac. annulatus* succumbed to netting damage sustained during initial capture and died during transportation back to the aquarium and were subsequently dissected for parasites upon arrival. The remaining *Ac. annulatus* rays were housed in the aquarium’s quarantine facility. After 24 hours, sampling of detritus from the bottom of the quarantine tank revealed the presence of monogenean eggs. Three rays were randomly removed for non-invasive parasitological examination using the method of Vaughan and Chisholm (2010a). Each of the three rays selected for the non-invasive treatment method was removed to a separate glass tank of 100 L volume, anaesthetised with 0.15 ml/L 2-phenoxyethanol, weighed, and given the anthelmintic praziquantel at 150 mg/kg by gavage (Vaughan and Chisholm 2010a). Thereafter, the volume of each 100 L tank was filtered through a 23 µm sieve to recover any monogeneans. The filtrate from each tank was placed into a separate glass inspection bowl with fresh, filtered seawater and observed under an Olympus SZ60 stereo zoom dissection microscope. Anaesthetised rays made a full recovery in separate holding tanks of fresh, filtered seawater. Live specimens of two unidentified species of Monocotylidae that originated from the gills, were recovered from the three treated, and one dissected *Ac. annulatus* rays; live specimens of two known representatives of Heterocotylinae were recovered from the gills of *Ae. Bovinus*: *Myliocotyle pteromylaei* Neifer, Euzet & Ben Hassine, 1999, and *Heliocotyle kartasi* Neifar, Euzet & Ben Hassine, 1999.

Monogeneans that were recovered from between the gill lamellae of the dissected *Ac. annulatus* ray, were initially observed alive in a glass inspection bowl containing fresh filtered seawater or individually on microscope slides in a drop of fresh, filtered seawater. Observations were recorded and photomicrographs taken of the live monogeneans. Thereafter, monogeneans were individually preserved flat in analytical reagent grade absolute ethanol (ARE). Some monogeneans were preserved unflattened in ARE for DNA extraction and for full or partial proteolytic digestion of haptoral armature and reproductive structures, following the methodology of Vaughan et al. (2008). Those for proteolytic digestion were rehydrated with freshwater and placed individually onto glass microscope slides. Their haptor was severed from the body-proper and treated separately to the body on the same slide before being mounted in glycerine jelly under a coverslip sealed with clear nail varnish. Flat-preserved monogeneans for staining were rehydrated in freshwater before being stained with either Alum Carmine or diluted Gormori’s Trichrome solutions, dehydrated in a graded ethanol series, cleared in Cedarwood oil, and permanently mounted individually in Canada balsam on glass microscope slides beneath a glass coverslip. Live monogeneans in temporary mounts of seawater, permanently mounted monogeneans and partial or total digests were examined using an Olympus CX 41 or Nikon Eclipse 200 compound light microscopes fitted with phase-contrast and dark-field optics. Photomicrographs were taken with an Olympus Altra 20 digital microscope camera mounted to the Olympus CX41 and drawings were made with the aid of a drawing tube. All measurements were taken using Olympus AnalySIS5 software calibrated to the Altra 20 and CX41. These measurements are given in micrometres as the mean ± standard deviation, followed in parenthesis by the range and the number of specimens measured.

DNA was extracted from two individual specimens of the two new species and from individuals of *Myliocotyle pteromyleai* Neifer, Euzet & Ben Hassine, 1999 (n = 1), *Heterocotyle tokoloshei* Vaughan & Chisholm, 2010 (n = 2), *Electrocotyle whittingtoni* Vaughan, Chisholm & Hansen, 2016 (n = 2) and *Neoheterocotyle robii* Vaughan & Chisholm, 2010 (n = 2) using the Mole DNA tissue kit on a MoleGenetics DNA extraction robot. PCR reactions were done using Illustra PuReTaq Ready-To-Go™ PCR Beads (GE Healthcare) in accordance with the manufacturer's instructions. Each reaction contained 1 μl of the forward primer, 1 μl of the reverse primer, 3μl of the template DNA and 20 μl of sterile water. The primer pair (C1 forward ACCCGCTGAATTTAAGCAT; D2 reverse TGGTCCGTGTTTCAAGAC) (Hassouna et al. 1984, see Chisholm et al. 2001a, b), was used to amplify a ∼950-bp fragment of the large subunit (LSU/28S) ribosomal DNA. A shorter fragment was also amplified using the primer combination Rob1 (Chisholm et al. 2001a) /D2. The PCR protocol was as follows: 4 min at 95 °C followed by 35 cycles of 1 min at 95°C, 1 min at 55°C, and 2 min at 72°C. PCR products were shipped to Macrogen Inc. (Seoul, Korea) for purification and sequencing on an Applied Biosystems 3730xl DNA Analyzer. All reactions were sequenced using the PCR primers. Sequence assembly and analysis of chromatograms were performed with Geneious Prime® version 2023.0.1 (www.geneious.com). All nucleotide sequence data were deposited in GenBank under accession numbers KT735368- KT735369 and OR351731-OR351739 (see Table 1). To reveal possible identity with other species present in GenBank, all obtained sequences were submitted to a BlastN search (Zhang et al. 2000) using default parameter settings.

**Table 1:** Taxa and their 28S rDNA sequence accession details used in the molecular phylogeny (new sequences in bold text)

Taxon	Host	Locality	Accession number	References
***Brancheocotyle imbricata* n. gen. et sp.**	***Acroteriobatus annulatus* (Smith, 1841)**	**Off Muizenberg, South Africa**	**OR351735, OR351736**	**Present study**
*Calicotyle affinis* Scott, 1911	*Chimaera monstrosa* Linnaeus, 1758	“Unknown fjord”, Norway	AF382061	Olson and Littlewood (2002)
*Cal.* cf*. kroyeri*	*Raja clavata* Linnaeus, 1758	North Sea	AF279744	Chisholm et al. (2001b)
*Cal.* cf*. kroyeri*	*Dipturus olseni* (Bigelow & Schroeder, 1951)	Gulf of Mexico	MW892410	Bullard et al. (2021)
*Cal. japonica* Kitamura, Ogawa, Shimizu, Kurashima, Mano, Taniuchi & Hirose, 2010	*Squalus mitsukurii* Jordan & Snyder, 1903	Japan	AB485996	Kitamura et al. (2010)
*Cal. kroyeri* Diesing, 1850	*Leucoraja naevus* (Müller & Henle, 1841)	Mediterranean Sea	AF279745	Chisholm et al. (2001b)
*Cal. kroyeri*	*Amblyraja radiata* (Donovan, 1808)	North Sea	AF279746	Chisholm et al. (2001b)
*Cal. kroyeri*	*R. radula* Delaroche, 1809	Mediterranean Sea	AF279747	Chisholm et al. (2001b)
*Cal. kroyeri*	*R.* sp*.* Linnaeus, 1758	South coast of Tasmania, Australia	AF279748	Chisholm et al. (2001b)
*Cal. palombi* Euzet & Williams, 1960	*Mustelus mustelus* (Linnaeus, 1758)	Tunisia, Mediterranean Sea	AF279749	Chisholm et al. (2001a)
*Cal. stossichi* Braun, 1899	*M. mustelus*	Tunisia, Mediterranean Sea	AF279751	Chisholm et al. (2001a)
*Cal.* sp. CWA1	*Urolophus viridis* McCulloch, 1916	South coast of Tasmania, Australia	AF279750	Chisholm et al. (2001b)
*Cal. urolophi* Chisholm, Beverley-Burton & Last, 1991	*U. cruciatus* (Lacepéde, 1804)	South coast of Tasmania, Australia	AF279752	Chisholm et al. (2001b)
*Cal. urolophi*	*U. paucimaculatus* Dixon, 1969	South coast of Tasmania, Australia	AF279753	Chisholm et al. (2001b)
*Capsala martinieri* Bosc, 1811	*Mola mola* (Linnaeus, 1758)	Skegness, England	AF382053	Olson and Littlewood (2002)
*Cathariotrema selachii* (MacCallum, 1916)	*Carcharhinus limbatus* (Valenciennes, 1839)	Gulf of Mexico	MW892404	Bullard et al. (2021)
*Cat. selachii*	*Sphyrna mokarran* (Rüppell, 1837)	Gulf of Mexico	MW892405	Bullard et al. (2021)
*Cat. selachii*	*Rhizoprionodon terraenovae* (Richardson, 1836)	Gulf of Mexico	MW892406	Bullard et al. (2021)
*Clemacotyle australis* Young, 1967	*Aetobatus narinari* (Euphrasen, 1790)	Heron Island, Australia	AF348350	Chisholm et al. (2001a)
*Decacotyle floridana* (Pratt, 1910)	*Ae. narinari*	Heron Island, Australia	AF348357	Chisholm et al. (2001a)
*Dec. lymmae* Young, 1967	*Ae. narinari*	Heron Island, Australia	AF348359	Chisholm et al. (2001a)
*Dec. tetrakordyle* Chisholm & Whittington, 1998	*Pastinachus sephen* (Fabricius, 1775)	Heron Island, Australia	AF348358	Chisholm et al. (2001a)
*Dendromonocotyle ardea* Chisholm & Whittington, 1995	*Pas. sephen*	Heron Island, Australia	AF348351	Chisholm et al. (2001a)
*Den. octodiscus* Hargis, 1955	*Hypanus americanus* (Hildebrand & Schroeder, 1928)	Gulf of Mexico	AF348352	Chisholm et al. (2001a)
*Dictyocotyle coeliaca* Nybelin, 1941	*Am. radiata*	North Sea	AF279754	Chisholm et al. (2001a)
*Di. coeliaca*	*Am. radiata*	North Sea	AF382062	Olson and Littlewood (2002)
*Di. coeliaca*	*R. montagui* Fowler, 1910	North Sea	AY157171	Lockyer et al. (2003)
***Electrocotyle whittingtoni* Vaughan, Chisholm & Hansen, 2010**	***Ac. annulatus***	**Off Miller’s Point, False Bay, South Africa**	**KT735368, KT735369**	**Present study**
*Empruthotrema aoneken* Irigoitia, Braicovich, Rossin & Timi in Irigoitia, Braicovich, Rossin, Canel, Levy, Farber & Timi, 2019	*Sympterygia bonapartii* Müller & Henle, 1841	Argentine Sea	MN190270, MN190272	Irigoitia et al. (2019)
*Em. aoneken*	*S. acuta* Garman, 1877	Argentine Sea	MN190271	Irigoitia et al. (2019)
*Em. dasyatidis* Whittington & Kearn, 1992	*Orectolobus maculatus* (Bonnaterre, 1788)	Heron Island, Australia	AF348345	Chisholm et al. (2001a)
*Em. dorae* Irigoitia, Braicovich, Rossin & Timi in Irigoitia, Braicovich, Rossin, Canel, Levy, Farber & Timi, 2019	*Myliobatis ridens* Ruocco, Lucifora, Díaz de Astarloa, Mabragaña & Delpiani, 2012	Argentine Sea	MN190273	Irigoitia et al. (2019)
*Em. dorae*	*Myliobatis goodei* Garman, 1885	Argentine Sea	MN190274	Irigoitia et al. (2019)
*Em. longipenis* Kritsky, Bullard, Ruiz & Warren, 2017	*Gymnura micrura* (Bloch & Schneider, 1801)	Gulf of Mexico	MW892409	Bullard et al. (2021)
*Em. orashken* Irigoitia, Braicovich, Rossin & Timi in Irigoitia, Braicovich, Rossin, Canel, Levy, Farber & Timi, 2019	*Atlantoraja castelnaui* (Miranda Ribeiro, 1907)	Argentine Sea	MN190264	Irigoitia et al. (2019)
*Em. orashken*	*Psammobatis bergi* Marini, 1932	Argentine Sea	MN190265	Irigoitia et al. (2019)
*Em. orashken*	*At. castelnaui*	Argentine Sea	MN190266	Irigoitia et al. (2019)
*Em. orashken*	*Ps. Normani* McEachran, 1983	Argentine Sea	MN190267	Irigoitia et al. (2019)
*Em. orashken*	*Ps. rudis* Günther, 1870	Argentine Sea	MN190268	Irigoitia et al. (2019)
*Em. orashken*	*Ps. normani*	Argentine Sea	MN190269	Irigoitia et al. (2019)
*Em. quindecima* Chisholm & Whittington, 1999	*Taeniura lymma* (Fabricius, 1775)	Heron Island, Australia	AF348346	Chisholm et al. (2001a)
*Entobdella hippoglossi* (Müller, 1776)	*Hippoglossus hippoglossus* (Linnaeus, 1758)	Stirling University, Scotland	AY486151	Whittington et al. (2004)
*Heterocotyle capricornensis* Chisholm & Whittington, 1996	*Pateobatis fai* (Jordan & Seale, 1906)	Heron Island, Australia	AF348360	Chisholm et al. (2001a)
***Het. tokoloshei*** Vaughan & Chisholm, 2010	***Bathytoshia brevicaudata* (Hutton, 1875)**	**Off Struisbaai, South Africa**	**OR351731, OR351732**	**Present study**
*Holocephalocotyle monstrosae* Derouiche, Neifar, Gey, Justine & Tazerouti, 2019	*Chimaera monstrosa* Linnaeus, 1758	Algeria, Mediterranean Sea	MN412655–MN412657	Derouiche et al. (2019)
*Loimopapillosum pascuali* Chero, Cruces, Sáez, Oliveira, Santos & Luque, 2021	*Hypanus dipterurus* (Jordan & Gilbert, 1880)	Off Puerto Pizarro, Tumbes, Peru	MZ367713, MZ367714	Chero et al. (2021)
*Loimos* sp. s LW-2021	Unknown	Unknown	OM060238	L. Wang (unpublished)
*Loimosina* sp. WAB-2014 (= *L. wilsoni*^1^)	*Sphyrna* sp.	Atlantic Ocean near Barra Velha, Brazil	KF908848	Boeger et al. (2014)
*Loimosina wilsoni* Manter, 1944^2^	*Sp. lewini* (Griffith & Smith, 1834)	Western North Atlantic Ocean, South Carolina, USA	OP348870– OP348872	Dalrymple et al. (2022)
*Mycteronastes icopae* (Beverley-Burton & Williams, 1989)	*Glaucostegus typus* (Anonymous [Bennett], 1830)	Heron Island, Australia	AF026113	Mollaret et al. (1997)
*Myc. icopae*	*G. typus*	Heron Island, Australia	AF348349	Chisholm et al. (2001a)
*Merizocotyle sinensis* Timofeeva, 1984	Unknown	Unknown	FJ514075	L. C. Su (unpublished)
*Monocotyle corali* Chisholm, 1998	*Pas. sephen*	Heron Island, Australia	AF348353	Chisholm et al. (2001a)
*Mon. helicophallus* Measures, Beverley-Burton & Williams, 1990	*Pat. fai*	Heron Island, Australia	AF348355	Chisholm et al. (2001a)
*Mon. multiparous* Measures, Beverley-Burton & Williams, 1990	*Pat. fai*	Heron Island, Australia	AF348356	Chisholm et al. (2001a)
*Mon. spiremae Measures, Beverley-Burton & Williams*, 1990	*Pat. fai*	Heron Island, Australia	AF348354	Chisholm et al. (2001a)
Monocotylidae sp. *incertae sedis*^3^	Unconfirmed	Heron Island, Australia	AF348362	Chisholm et al. (2001a)
***Myliocotyle pteromylaei* Neifer, Euset & Ben Hassine, 1999**	***Aetomylaeus bovinus* (Geoffroy Saint-Hilaire, 1817)**	**Off Muizenberg, South Africa**	**OR351739**	**Present study**
*Neoheterocotyle quadrispinata* Nitta, 2019	*Rhinobatos hynnicephalus* Richardson, 1846	Hiroshima, Japan	LC428038	Nitta (2019)
*N.* sp.^4^	*G. typus*	Heron Island, Australia	AF026107	Mollaret et al. (1997)
*N. rhinobatidis* (Young, 1967)	*G. typus*	Heron Island, Australia	AF348361	Chisholm et al. (2001a)
*N. rhynchobatis* (Tripathi, 1959)	*G. typus*	Heron Island, Australia	AF348363	Chisholm et al. (2001a)
***N. robii*** Vaughan & Chisholm, 2010	***Acroteriobatus annulatus***	**Off Cape Agulhas, South Africa**	**OR351737, OR351738**	**Present study**
*Peruanocotyle pelagica* Ruiz-Escobar, Torres-Carrera & Ramos-Sánchez in Ruiz-Escobar, Torres-Carrera, Ramos- Sánchez, García-Prieto, Mendoza-Garfias & Oceguera-Figueroa, 2022	*Rhinoptera steindachneri* Evermann & Jenkins, 1891	Off Oaxaca, and Guerrero, Mexico	OK018174	Ruiz-Escobar et al. (2022)
*Potamotrygonocotyle aramasae* Domingues, Pancera & Marques, 2007	Unknown	‘Araguaia’, Brazil	FJ755804	Fehlauer-Ale and Marques (unpublished)
*Po. aramasae*	*Paratrygon aiereba* (Walbaum, 1792)	Tocantins River, Amazonas Basin, Brazil	FJ755805	Fehlauer-Ale and Littlewood (2011)
*Po. aramasae*	*Par. aiereba*	Rio Negro River, Amazonas Basin, Brazil	FJ755806	Fehlauer-Ale and Littlewood (2011)
*Po. aramasae*	*Par. aiereba*	Yavari River, Amazonas State, Amazonas Basin, Brazil	JN379514	Fehlauer-Ale and Littlewood (2011)
*Po. chisholmae* Domingues & Marques, 2007	*Potamotrygon brachyura* (Günther, 1880)	Uruguay River, Rio Grande do Sul State, La Plata Basin, Brazil	JN379519	Fehlauer-Ale and Littlewood (2011)
*Po. dromedarius* Domingues & Marques, 2007	*Pot.* sp.	Parana River, Tocantins State, Amazonas Basin, Brazil	JN379517	Fehlauer-Ale and Littlewood (2011)
*Po. dromedarius*	*Pot. Motoro* (Müller & Henle, 1841)	Salobra River, Mato Grosso do Sul State, La Plata Basin, Brazil	JN379518	Fehlauer-Ale and Littlewood (2011)
*Po. quadracotyle* Domingues, Pancera & Marques, 2007	*Pot.* sp.	Rio Negro River, Amazonas Basin, Brazil	FJ755807	Fehlauer-Ale and Littlewood (2011)
*Po. rarum* Domingues, Pancera & Marques, 2007	*Pot. Schroederi* Fernández-Yépez, 1958	Rio Negro River, Amazonas Basin, Brazil	FJ755809	Fehlauer-Ale and Littlewood (2011)
*Po. rionegrense* Domingues, Pancera & Marques, 2007	*Pot. motoro*	Rio Negro River, Amazonas Basin, Brazil	FJ755810	Fehlauer-Ale and Littlewood (2011)
*Po. tocantinsense* Domingues & Marques, 2011	*Pot.* cf*. scobina* Garman, 1913	Tocantins River, Amazonas Basin, Brazil	FJ755811	Fehlauer-Ale and Littlewood (2011)
*Po. tsalickisi* Mayes, Brooks & Thorson, 1981	*Pot.* cf*. falkneri* Castex & Maciel, 1963	Salobra River, La Plata Basin, Brazil	JN379513	Fehlauer-Ale and Littlewood (2011)
*Po. umbella* Domingues, Pancera & Marques, 2007	*Pot* sp.	Rio Negro River, Amazonas Basin, Brazil	FJ755808	Fehlauer-Ale and Littlewood (2011)
***Scuticotyle cairae* n. gen. et sp.**	***Ac. annulatus***	**Off Muizenberg, South Africa**	**OR351733, OR351734**	**Present study**
*Thaumatocotyle australensis* Beverley-Burton & Williams, 1989	*Pat. fai*	Heron Island, Australia	AF348348	Chisholm et al. (2001a)
*Th.* sp.	*Bathytoshia centroura* (Mitchill, 1815)	Gulf of Mexico	MW892408	Bullard et al. (2021)
*Th. urolophi* (Chisholm & Whittington, 1999)	*U. bucculentus* Macleay, 1884	Tasmania, Australia	AF348347	Chisholm et al. (2001a)
*Troglocephalus rhinobatidis* Young, 1967	*G. typus*	Heron Island, Australia	AF026110	Mollaret et al. (1997)
*Tr. rhinobatidis*	*G. typus*	Heron Island, Australia	AF348364	Chisholm et al. (2001a)

^1^Dalrymple et al. (2022) considered *Loimosina* sp. WAB-2014 of Boeger et al. (2014) to be representative of *L. wilsoni*

^2^Dalrymple et al. (2022) suggested that *L. parawilsoni* Bravo-Hollis, 1970 is a junior synonym of *L. wilsoni*

^3^Considered in the present study as Monocotylidae sp. *incertae sedis* but originally accessioned as *Ne. rhinobatis* by Chisholm et al (2001a); host species also considered unconfirmed

^4^Considered in the present study as nonugen but originally accessioned as *N. rhinobatidis* byMollaret et al. (1997)

The phylogenetic analyses included the new sequences obtained in the present study in addition to all sequences taken from nominal species of Monocotylidae that were available (Table 1). Sequences of *Capsala martinieri* Bosc, 1811 (accession number AF382053), *Entobdella hippoglossi* (Müller, 1776) (accession number AY486151) Blainville, 1818, and *Benedenia lutjani* Whittington & Kearn, 1993 (accession number AY033939), were used as the outgroup, which is the same used by Bullard et al. (2021). For species for which several identical sequences were available, only the longest of these identical sequences was included. In addition, some sequences retrieved from GenBank were evaluated to be too short to be included (e.g., *Triloculotrema* sp. AF387512, see Bullard et al., 2021). The final selection consisted of 79 unique sequences representing 62 species, including the outgroup. Alignment of the selected SSU sequences was constructed using MAFFT v7 online (L-INS-I algorithm) (Katoh et al. 2019), resulting in a final alignment of 1236 nucleotide sites (Supplementary file S1). Maximum-likelihood (ML) phylogenetic trees of the alignment were constructed with W-IG-TREE (Trifinopoulos et al. 2016) accessible from http://iqtree.cibiv.univie.ac.at. W-IG-TREE automatically determined the best-fit substitution models to be GTR+F+I+G4 according to the Bayesian information criterion. Branch support was assessed by ultrafast bootstrap (UFBoot2; Hoang et al. 2018) with the number of bootstrap alignments and the maximum number of iterations set to 10000. For Ultrafast bootstrap support, we consider values of 95% or higher as statistically significant and indicative of a well-supported group, while those with lower values are not considered significant. Finally, the sequence alignment was also processed with MEGAX (Stecher et al. 2020) to calculate the number of pairwise base differences (Supplementary file S2).

Specimens of *C. selachii*, *Het. pastinacae*, *Hel. kartasi* and *My. pteromylaei* were identified using Bullard et al. (2021), Neifar et al. (1998; 1999a, b) or Chisholm (1995). The type series and vouchers of both new taxa described herein, the vouchers of *C. selachii*, and three vouchers of *Het. pastinacae* are deposited in the Australian Helminthological Collection (AHC) at the South Australian Museum in Adelaide, South Australia, Australia. Vouchers of *Hel. kartasi* and *My. pteromylaei*, and one voucher of *Het. pastinacae* are deposited in the Iziko South African Museum, Cape Town, South Africa (SAMC). The following museum specimens were examined for comparative purposes: British Museum of Natural History, *Mehracotyle insolita* (BMNH 2001.8.6, three paratypes); The Smithsonian Institute, *Anoplocotyloides chorrillensis* Luque & Iannacone, 1991 (USNM 1376670/USNPC 081339, holotype, high resolution Z-stack imagery; USNM 1376671/USNPC 81340, paratype), *Troglocephalus rhinobatidis* (USNPC 61753, paratype); Oswaldo Cruz Institute helminth collection, Brazil, *Peruanocotyle chisholmae* (CHIOC 39080a–c, high-resolution photomicrographs of three paratypes); South Australian Museum, *Tr. rhinobatidis* (AHC 36754–6, seven vouchers); high resolution photomicrograph library of Professor Marcus Vinicius Domingues for *Anoplocotyloides papillatus* (Harold W. Manter Laboratory of Parasitology Collection [HWML] 31181; USNPC 61040), *Dasybatotrema dasybatis* (HWML 17119_17164; USNPC 35655 [paratype], 35657–58), *Heliocotyle kartasi* (Muséum national d'Histoire naturelle, Paris, France [MNHN] 580 hf [paratype]), *Mehracotyle insolita* (MNHN 36 HG tg 176 [paratype]), *Myliocotyle pteromylaei* (MNHN 644 HF Tk 178, 645 HF Tk 180 [paratypes]), *Neoheterocotyle impristi* (USNPC 77301, 38159 [holotype]), *Nonacotyle pristis* (Meguro Parasitological Museum, Tokyo [MPM] 19550 [holotype]), *Spinuris lophosoma* (HWML 17374, 31182–3; USNPC 47835 [holotype]), *Timofeevia rajae* (USNPC 84479).

## Results

**Monocotylidae Taschenberg, 1879.**

**Cathariotrematinae Bullard in Bullard, Warren & Dutton,** **2021.**

*Cathariotrema selachii* (MacCallum, 1916).

Material deposited: AHC 37073 (2 vouchers).

Host: *Galeorhinus galeus* (Linnaeus), male, 120 cm total length.

Microhabitat: nasal fossae.

Locality: Off Cape Agulhas, South Africa, trawl number A28414.

Collection date: 13 April 2008.

### Remarks

The South African locality for *C. selachii* is a new locality record for this species.

**Heterocotylinae** Chisholm, Wheeler & Beverley-Burton, 1995.

Revised diagnosis. Anterior head region rounded, with pairs of few (usually three), small anterolateral gland-duct openings on the anterolateral margin, and usually one pair of smaller, indistinct anteromedial gland-duct openings near the anterior margin. Two anterolateral and one anteromedial gland usually present in the head region. Ventral pits absent. Eyespots present or absent. Pharynx muscular, ovoid. Intestinal caeca non-diverticular, non-confluent posteriorly. Ovary simple, positioned anterior to testis, ovarian branch loops right intestinal caecum. Testis single, ovoid, tapering posteriorly, or lobed. Vaginal pore unarmed. Vagina single, with sclerotised spines internally, sclerotised walls, or completely unsclerotised. Seminal receptacle single, ovoid, or elongated. Musculo-glandular ejaculatory bulb present, connected to sclerotised male copulatory organ, with or without accessory piece. Common genital pore unarmed. Haptor roughly circular with one central and four, seven or eight peripheral loculi. Marginal membrane present. Pair of hamuli present, generally C-shaped as a function of well-developed, terminally rounded superficial root; terminally truncated in *Electrocotyle* Vaughan, Chisholm & Hansen, 2016. Deep root usually short, narrow, but elongated and broad in *Electrocotyle*. Fourteen marginal hooks distributed in the marginal membrane. Single straight, slightly sinuous, or sinuous, or double-sinuous septal ridge present. Numerous longitudinal sinuous ridges on ventral surface of peripheral loculi present or absent. Marginal haptoral papillae absent. Septal sclerites absent. Rounded dorsal accessory structure(s), either unsclerotised, or with sclerotised rounded edge present on dorsal surface of posterior loculus or posterior and posterolateral loculi. Gill parasites of myliobatiform stingrays and torpediniform electric rays.

Type-genus: *Heterocotyle* Scott, 1904.

Other subordinate taxa (monotypic genera indicated by their species): *Potamotrygonocotyle* Mayes, Brooks & Thorson, 1981, *Heliocotyle* Neifar, Euzet & Ben Hassine, 1999, *Myliocotyle* Neifar, Euzet & Ben Hassine, 1999, *Malalophus jensenae* Chisholm & Whittington, 2009, *Septesinus gibsoni* Chisholm, 2013, *E. whittingtoni* Vaughan, Chisholm & Hansen, 2016.

*Heterocotyle pastinacae* Scott, 1904.

Material deposited: AHC 35116 (3 vouchers); SAMC-A029475 (1 voucher).

Host: *Dasyatis chrysonota* (Smith), two female, disk width = 68 cm; 80 cm; one male, disk width = 56 cm.

Microhabitat: gill lamellae.

Locality: Off Cape Agulhas, South Africa, trawl numbers A28425, A28432, and A28426, respectively.

Collection dates: 15 April, 16 April, and 15 April 2008, respectively.

*Heliocotyle kartasi* Neifar, Euzet & Ben Hassine, 1999.

Material deposited: SAMC-A029489 (1 voucher).

*Myliocotyle pteromylaei* Neifer, Euzet & Ben Hassine, 1999.

Material deposited: SAMC-A029488 (1 voucher).

Host: *Aetomylaeus bovinus* (Geoffroy Saint-Hilaire), female.

Microhabitat: gill lamellae.

Locality: Muizenberg beach (34°06’13” S, 18°29’00” E), False Bay, South Africa.

Collection date: February 2010.

### Remarks

The Heterocotylinae was originally proposed by Chisholm et al. (1995) to accommodate all monocotylids with one central and seven, eight or nine peripheral haptoral loculi that possess at least four sclerotised (or partially sclerotised) dorsal haptoral structures on the posterior and posterolateral loculi. Subsequently, the genera *Heliocotyle* and *Malalophus* were included, with members that have a single, rounded, partially sclerotised dorsal haptoral accessory structure on the posterior loculus (Neifar et al. 1999a; Chisholm and Whittington 2009). Domingues et al. (2007) included a species of *Potamotrygonocotyle* with only four peripheral haptoral loculi, which appears to have been missed in most subsequent treatments that included the subfamily diagnosis, even to the most recent. Later, Vaughan et al. (2016) added *E. whittingtoni*, amending the subfamily to include four non-sclerotised dorsal haptoral accessory structures.

Bullard et al. (2021) recently proposed three morphological groups for the Heterocotylinae genera. Group 1 included *Electrocotyle*, *Heterocotyle*, *Myliocotyle*, *Potamotrygonocotyle*, and *Spinuris*. This group consisting of those representatives with one central and eight peripheral loculi – two of which are interhamular, four “flaps” (= dorsal haptoral accessory structures) dorsal to the four posterior-most loculi, the absence of ventral locular-surface ridges, and the presence of a septal ridge in all but *Spinuris*. Group 2 included *Neoheterocotyle*, *Septisinus* [sic; = *Septesinus*], and *Nonacotyle*, with one central and seven peripheral loculi – one interhamular, three “flaps” dorsal to the three posterior-most loculi, the absence of ventral locular-surface ridges and septal ridges. Group 3 included *Malalopholus* [sic; = *Malalophus*] and *Heliocotyle*, with one central and seven peripheral loculi, one “flap” dorsal to the posterior loculus, the presence of ventral locular-surface ridges, and the absence of a septal ridge. Unfortunately, these groups are inaccurate. The first group excluded *Po. quadracotyle* Domingues, Pancera & Marques, 2007 with one central and four peripheral loculi, where a pair of dorsal haptoral accessory structures are present on the single interhamular posterior loculus, and a single bilobed dorsal haptoral accessory structure is present on each of the two lateral loculi (Domingues et al. 2007). Group 1 also excluded *Po. septemcotyle* Domingues & Marques, 2011, which has seven peripheral loculi (Domingues and Marques 2011). *Nonacotyle* in the second group has nine peripheral loculi, not seven, and has six dorsal haptoral accessory sclerites, two dorsal to the posterior loculus, and two dorsal to the two posterolateral loculi; *Neoheterocotyle* species have four or six dorsal haptoral accessory sclerites, two dorsal to the posterior loculus, and one or two dorsal to the two posterolateral loculi. *Septesinus* has four dorsal haptoral accessory structures, two on the posterior loculus, and one on both posterolateral loculi. All genera in groups 2 and 3 have a present septal ridge.

**Fig. 1:**
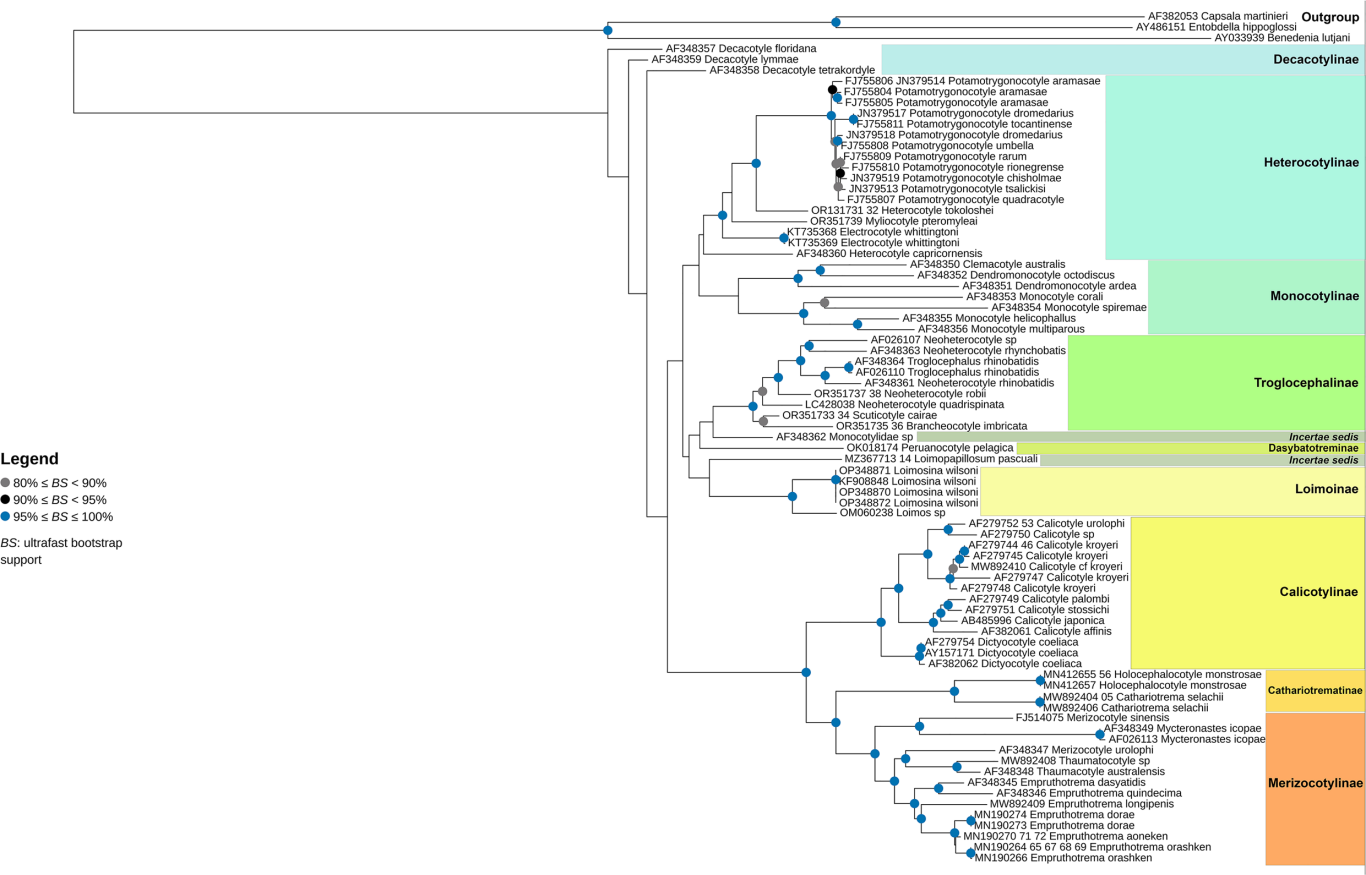
Molecular phylogenetic analyses of the Monocotylidae assessed by maximum likelihood inference. The best fit tree is shown. Branch support assessed by ultrafast bootstrap (UFBoot2).

The monophyly of the Heterocotylinae, excluding *Het. capricornensis*, is well supported in our phylogeny (Fig. 1), which includes new sequences for *Het. tokoloshei* and *My. pteromylaei*. *Heterocotyle* is currently paraphyletic, but there are only two representatives for which we currently have molecular data. Additional sequences of *Heterocotyle* but also currently unrepresented taxa, *Heliocotyle*, *Malalophus* and *Septesinus* are required to provide a greater understanding of the evolutionary relationships within this group.

Heterocotylinae is amended in exclusion of *Neoheterocotyle* Hargis, 1955, *Spinuris* Doran, 1953, and *Nonacotyle pristis* Ogawa, 1991, all of which have prominently projecting, often spiculate dorsal haptoral sclerites. This exclusion also sees the removal of taxa with numerous anterior gland-duct openings, and those with four pairs of ventral pits in the anterior head region. The amended subfamily includes members with (a) rounded non- or partially sclerotised dorsal haptoral accessory structure(s) from myliobatiform stingrays and torpediniform electric rays and excludes monocotylids of rhinopristiform shovelnose rays.

*Dasyatis chrysonota* from South Africa is a new host record for *Het. pastinacae*. The discovery of *Het. pastinacae* on *D. chrysonota* rejects the hypothesis of Neifar, Euzet and Ben Hassine (2000) that *Heterocotyle* species are strictly host-specific and that they can be used to discriminate host species. Bullard et al. (2021) referred to the putative host-specificity dogma, evident in the monocotylid literature, suggesting that host-specificity in the family is probably less strict than has previously been considered. Indeed, this dogma is merely the result of the lack of sampling resolution across an extensive potential host diversity, much of which remains unexplored. The South African localities for *Het. pastinacae*, *Hel. kartasi*, and *My. pteromylaei* constitute new locality records for these species.

**Troglocephalinae Vaughan n. subfam.**

Diagnosis. Anterior head region arrow-shaped or rounded, with numerous single, distinct gland-duct openings along its anterolateral and lateral margins, not continuous along its entire posterior margin. Gland-duct openings connected to glands via network of inconspicuous gland ducts. Two anterolateral and one anteromedial gland usually present in the head region. Four pairs of ventral pits present in anterior head region. Eyespots present. Pharynx muscular, round, or ovoid. Intestinal caeca non-diverticular, non-confluent posteriorly. Ovary simple or lobed, positioned anterior to, or ventrally over single large ovoid, round, or lobed testis. Ovarian branch loops or does not loop right intestinal caecum. Vaginal pore armed or unarmed. Vagina single, with sclerotised or unsclerotised walls. Seminal receptacle single or bipartite. Oötype with ascending limb. Seminal vesicle a simple inflation from vas deferens. large musculo-glandular ejaculatory bulb present. Sclerotised male copulatory organ with or without accessory piece, with or without muscular sheath, without accessory filament; male copulatory organ significantly reduced in some species. Common genital pore armed or unarmed. Haptor roughly circular with one central and seven, eight or nine peripheral loculi. Marginal membrane present. Pair of hamuli present, variable in shape, with or without accessory piece. Fourteen marginal hooks distributed in the marginal membrane. Single septal ridge present or absent, sinuous, zigzag, or straight. Marginal haptoral papillae absent. Septal sclerites absent. Prominent, projecting dorsal accessory sclerites present or absent; when present, dorsal on the posterior and posterolateral loculi. Mature sperm with two normal axonemes, ¾ microtubule ring, microtubule ornamentation, distal microtubule location. Gill parasites of rhinopristiform shovelnose rays.

Type-genus: *Troglocephalus rhinobatidis* Young, 1967.

Other subordinate taxa (monotypic genera indicated by their species): *Spinuris* Doran, 1953, *Neoheterocotyle* Hargis, 1955, *Anoplocotyloides* Young, 1967, *Nonacotyle pristis* Ogawa, 1991, *Mehracotyle insolita* Neifar, Euzet & Ben Hassine, 2002, *Scuticotyle cairae* **n. gen. et sp.**, and *Brancheocotyle imbricata* **n. gen. et sp.**

ZooBank registration: urn:lsid:zoobank.org:act:B6605621-6382-42A2-8114-0F44DFB3F2CB.

### Remarks

*Troglocephalus* was chosen as the type-genus for the new subfamily because it was the original representative taxon to be excluded from all known subfamilies and was relegated to enigmatic status in the revised classification of the family by Chisholm et al. (2001a). The proposal of the new subfamily sees the inclusion of *Anoplocotyloides* and *Mehracotyle insolita*, transferred from Dasybatotreminae, *Spinuris*, *Neoheterocotyle* and *Nonacotyle pristis* from Heterocotylinae, and the inclusion of *Troglocephalus rhinobatidis* (previously *incertae sedis*). The current molecular phylogeny supports the separation of the representatives of the subfamily that have been sequenced, from Heterocotylinae, and from *P. pelagica*, the sole representative of Dasybatotreminae, further supporting the morphological hypothesis for the subfamily (Fig. 1). Currently, *Anoplocotyloides*, *Mehracotyle*, *Nonacotyle*, *Timofeevia*, and *Spinuris* are not represented in the molecular phylogeny, which is unavoidable and unfortunate, because a greater understanding of the relationships between these troglocephalines will ultimately result from the inclusion of their representative sequences. All these troglocephaline species are united by the morphology of the anterior head region and are currently only known from rhinopristiform shovelnose rays.

Some minor historic errors exist in the literature regarding the accuracy of some morphological features of representatives of the new subfamily. For example, Vaughan and Chisholm (2010b) illustrated and described what they considered the anterolateral glands in *Ne. robii* Vaughan & Chisholm, 2010; however, these illustrated structures are likely ganglia associated with anterior sensory cells, given that they do not ‘open’ to the ventral surface of the haptor as gland-duct openings do. The sub-anterolateral and lateral glands, depicted as close together in this species, are likely not separate, and are the true anterolateral glands. Nitta (2019) labelled the anterior gland-duct openings along the margin of *Ne. quadrispinata* Nitta, 2019 as the anteromedian gland, which is a misnomer, where similarly, the anterior gland-duct opening of the anterior head glands were historically referred to as head organs; they are neither glands or organs (see Chisholm and Whittington 1996 for discussion). Chero et al. (2018) discussed *Anoplocotyloides* species as having marginal haptoral papillae. These marginal haptoral papillae are extensions from the outer-ring septum of the haptor (see Chisholm et al. 1995), and are only present in *Clemacotyle*, *Dendromonocotyle* and *Monocotyle* species (Monocotylinae), *Dasybatotrema* species and *P. pelagica* (Dasybatotreminae). The ‘marginal haptoral papillae’ considered by Chero et al. (2018) may stem from the descriptions of *Anoplocotyloides chorrillensis* Luque & Iannacone, 1991, which included small, rounded extensions of the marginal membrane that accommodate the 14 marginal hooks as marginal papillae. Given the definition of this character by Chisholm et al. (1995), marginal haptoral papillae are absent in *Anoplocotyloides* (see also Young 1967). The presence of an accessory filament on the male copulatory organ for *A. papillatus* (see Chisholm et al. 1995) is erroneous, possibly originating from the mistranslation of Bravo-Hollis (1969), where the male copulatory organ is described as surrounded by a delicate tubular membrane. No mention of an accessory filament is given in Doran (1953), Bravo-Hollis (1969), or Young (1967). Observations of the four pairs of ventral pits on the anterior head region were unclear in *Anoplocotyloides chorrillensis* and *Spinuris* species due to the level of staining employed, or the quality of presentation of this region on the slides; however, this character is clearly visible in *A. papillatus*, and the remaining characters of the new subfamily warrant the inclusion of *Spinuris* species.

Including the two new species into the original Dasybatotreminae classification would have resulted in yet another amendment of that subfamily to include additional ambivalent character states and the avoidance of resolving clear issues in Dasybatotreminae and Heterocotylinae. This would have resulted in Dasybatotreminae, as originally classified, becoming a dumping ground for clearly morphologically diverse taxa not accommodated in other subfamilies.

***Scuticotyle*** **n. gen.**

Generic diagnosis. Anterior region of body with three glands; anteromedian gland and pair of anterolateral glands. Numerous, singular, large, pad-like marginal anterior gland-duct openings present. Four pairs of anterior ventral pits present. Paired anterior dorsal pits present. Granulated eyespots present. Haptor roughly circular with one central, and seven peripheral loculi of approximately equal size. Septal ridge absent. Haptoral papillae absent. Septal or dorsal haptoral accessory sclerites absent. Single large conspicuous cells associated with junction of inner ring septum and radial septa. Marginal membrane present. Hamuli with well-developed superficial root; without accessory piece. Fourteen marginal hooklets distributed in the marginal membrane. Posterior-most portion of body proper forms shield-like structure over dorsal portion of haptor. Testis ovoid, medial. Musculo-glandular ejaculatory bulb present. Male copulatory organ sclerotised, reduced, without accessory piece, accessory filament, or muscular sheath. Ovary not lobed; ovarian branch loops around right intestinal caecum. Caecum without diverticula; non-confluent posteriorly. Unarmed vagina as a simple tube; vaginal walls not sclerotised. Field of papillae present on ventral tegument in area between vagina and seminal vesicle, either side of vas deferens. Oötype ascending limb curved, looped or straight. Common genital pore unarmed. Gill parasites of Rhinobatidae Bonaparte, 1835.

Etymology: Named for the shield-like structure protecting the haptor, after the Latin for shield, *scutus*.

Type and only species: *Scuticotyle cairae* **n. sp.**

ZooBank registration: urn:lsid:zoobank.org:act:857AB2B8-E9CB-40A5-B585-3107AB55FAE5

**Fig. 2:**
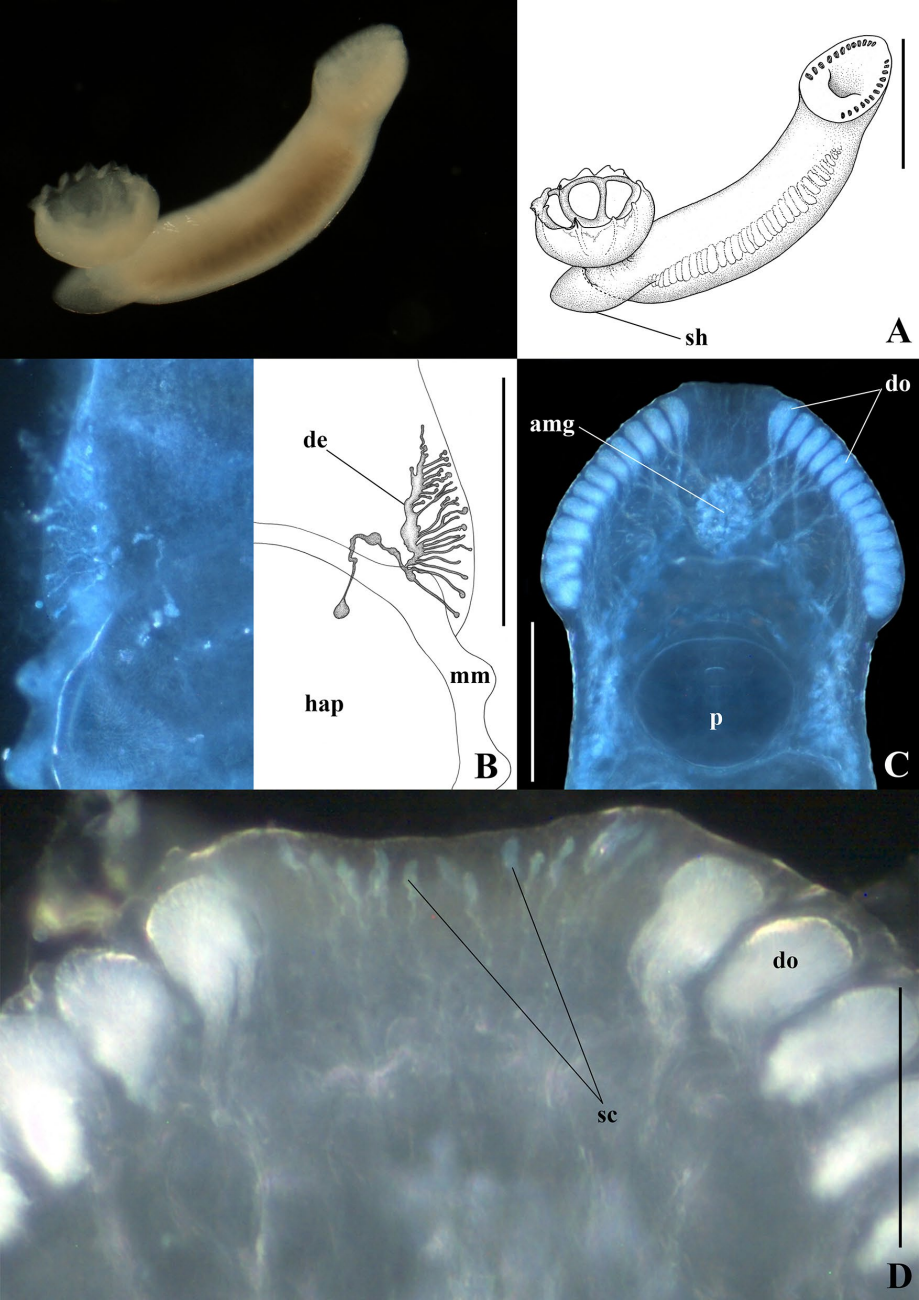
Photomicrographs and representative illustrations of live specimens of *Scuticotyle cairae* **n. gen. et sp.**; A, whole worm; B, corresponding sides of posterior part of body; C, anterior head region; D, close-up of anterior-most part of head region. Abbreviations: amg, anteromedian gland; de, bilateral dendritic structures of unconfirmed function; do, duct openings; hap, haptor; mm, marginal membrane; p, pharynx; sc, sensory cell structures; sh, shield. Scale bars: A = 600 μm, B, C = 300 μm, D = 100 μm.

**Fig. 3:**
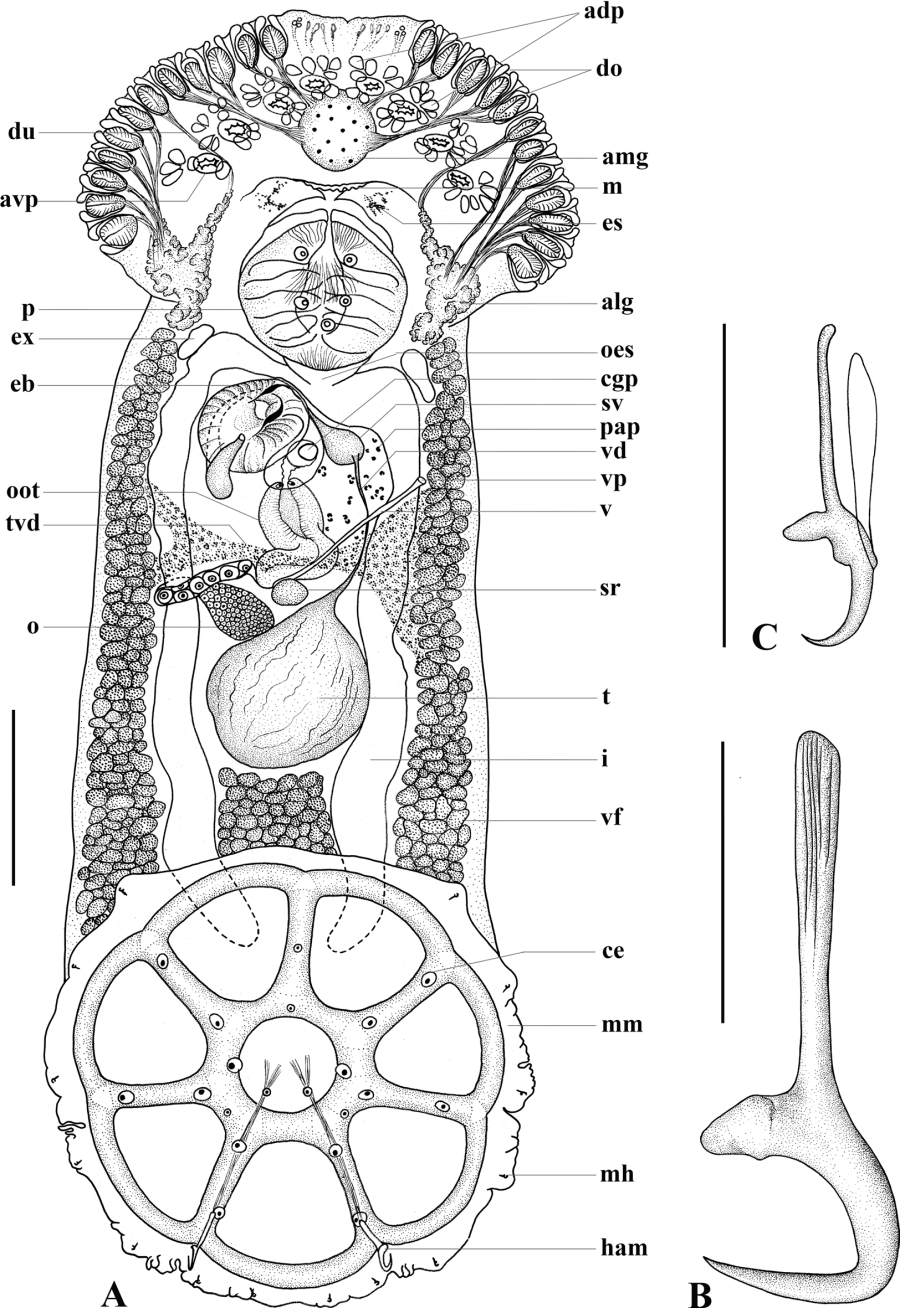
*Scuticotyle cairae* **n. gen. et sp.**; A, whole mount ventral view; B, hamulus; C, marginal hooklet. Abbreviations: adp, anterior dorsal pits; alg, anterolateral gland; avp, anterior ventral pit; ce, large cell of unknown function; cgp, common genital pore; du, ducts; eb, ejaculatory bulb with sclerotised walls; es, eyespots; ex, excretory bladder; ham, hamulus; i, intestinal caecum; m, mouth; mh, marginal hooklet; o, ovary; oes, oesophagus; oot, oötype; pap, field of papillae; sr, seminal receptacle; sv, seminal vesicle; t, testis; tvd, transverse vitelline duct; v, vagina; vd, vas deferens; vf, vitelline follicle; vp, vaginal pore. Other abbreviations as for Fig. 1. Scale bars: A = 200 µm, B = 40 µm, C = 12 µm.

**Fig. 4:**
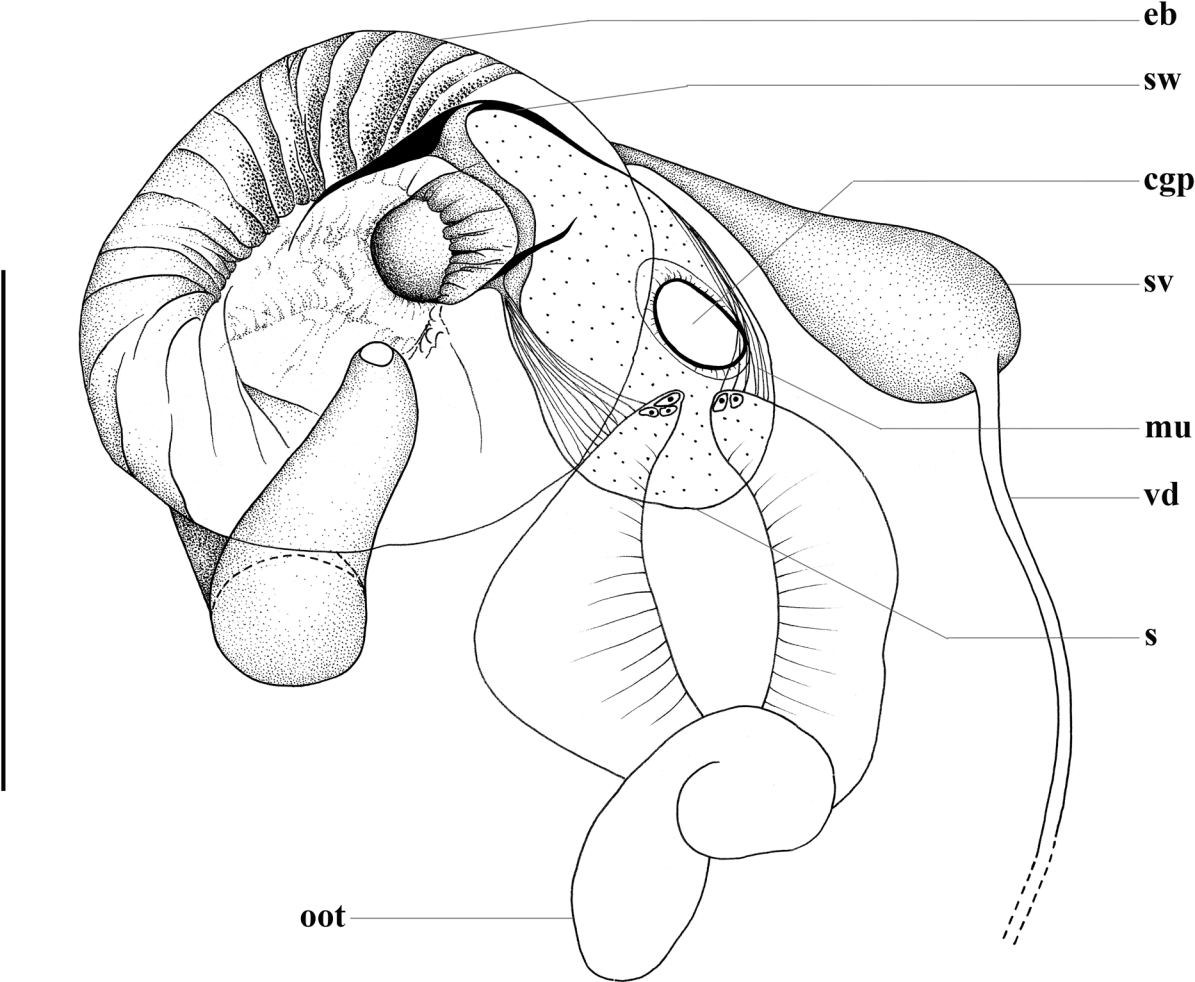
*Scuticotyle cairae* **n. gen. et sp.** Reproductive structures. Abbreviations: mu, muscles; s, common space between oötype and sclerotised walls of ejaculatory bulb; sw, sclerotised walls of ejaculatory bulb. Other abbreviations as for Fig. 2. Scale bar = 200 µm.

***Scuticotyle cairae* n. sp.** (Figs 2–4)

Description. Based on the flat-preserved whole-mounted holotype and 25 paratypes, 3 digests, and observations made from live specimens. Total body (excluding haptor) 1716 ± 256.1(1406–2225, n = 26) long, 560 ± 104.4(420–850, n = 26) wide at widest point. Posterior part of body-proper forming distinct protective shield over dorsal region of haptor, observed in live specimens (Fig. 2A). Pair of bilateral dendritic structures of unconfirmed function, possibly sensory, visible only in live specimens, associated with posterior part of body proper (Fig. 2B). Haptor subterminal, roughly circular, 571 ± 61.0(442–701, n = 23) long, 613 ± 61.2(518–716, n = 23) wide; divided into one central and seven deep peripheral loculi (Fig. 3A). Septal ridge absent. Marginal membrane present. Hamuli present at junction of posterior-most radial septa and outer-ring septum (Fig. 3A). Hamulus total length 84 ± 3.4(79–88, n = 6) with well-developed superficial root and thin, long deep root; accessory piece absent (Fig. 3B). Terminus of hamulus deep root attached to conspicuous muscle, originating from central loculus (Fig. 3A). Fourteen marginal hooklets with narrow handle and domus 13 ± 0.4(12–14, n = 15) long distributed in the marginal membrane as illustrated (Figs 3A, C). Single, conspicuous nucleated cells of unknown function associated with junction of inner-ring septum and radial septa, outer portion of radial septa, junction of hamulus deep root and musculature, and within central loculus where muscle fibres splay (Fig. 3A). Mouth ventral, subterminal. Broad anterior head region with 12 or 13 pairs of conspicuous gland-duct openings with ventral pad-like termini situated marginally (Fig. 2C, D, 3A). Numerous sensory cell structures associated with anterior-most end of head region, between anterior-most pair of gland-duct openings, not connected to any glands (Figs 2D, 3A). Four pairs of ventral pits present, flanked dorsally by pairs of smaller ovoid pits each opening separately to dorsal surface (Fig. 3A). Three anterior glands present (Fig. 3A), type of secretions not observed. Anteromedian gland circular, positioned medially, anterior to mouth (Fig. 3A). Two pairs of fine ducts lead laterally from anteromedian gland travelling between anterior ventral pits; anterior-most pair splits to service two anterior-most pairs of marginal duct openings; posterior pair of fine ducts splits into three to service next consecutive row of three pairs of marginal duct openings (Fig. 3A). Pair of anterolateral glands on either side of pharynx with fine ducts leading anteriorly; single duct travelling between posterior-most pair of ventral pits to service single median lateral duct opening; six or seven remaining ducts travelling directly to remaining pairs of six or seven marginal duct openings (Fig. 3A). Pharynx (Figs 2C, 3A) large, ovoid, muscular, 215 ± 30.1(170–263, n = 26) long, 248 ± 37.7(185–334, n = 26) wide with two or three pairs of conspicuously nucleated cells similar to those of haptor. Short oesophagus leads to laterally bifurcating intestinal caecum; excretory bladders adjacent to initial anteriorly directed curves in caecal branches (Fig. 3A). Both parallel caecal branches without diverticula, non-confluent, ending in posterior portion of body proper in line with anterior half of haptor (Fig. 3A). Eyespots in form of dispersed pigment granules anterodorsally to pharynx, either side of mouth (Fig. 3A). Ovoid testis 173 ± 26.2(139–263, n = 26) long, 188 ± 38.3(130–304, n = 26) wide (Fig. 3A). Narrow vas deferens arises from anterior portion of testis, travelling left, dorsal, and anterior to vagina before swelling abruptly left of common genital pore to form seminal vesicle (Figs 3A, 4). Ventral tegument in area either side of vas deferens between vagina and seminal vesicle with numerous small papillae (Fig. 3A). Seminal vesicle narrows dorsally at anterior portion of ejaculatory bulb, curves posteriorly, traveling dorsal over ejaculatory bulb, widening distally before sharply curving ventrally and anteriorly, entering ejaculatory bulb near its centre (Figs 3A, 4). Ejaculatory bulb roughly circular, musculo-glandular, 142 ± 19.8(110–184, n = 25) in diameter. Male copulatory organ reduced to short, raised, thinly sclerotised adjacent walls, exiting centre of ejaculatory bulb, linking up directly with common space surrounding unarmed common genital pore (Fig. 4). Longitudinal muscles associated with thin sclerotised walls (Fig. 4) but not forming muscular sheath. Ovary small, oval 79 ± 21.0(55–126, n = 25) long, 108 ± 27.0(66–162, n = 25) wide positioned immediately anterior and right of testis (Fig. 3A). Right ovarian branch loops caecum dorsoventrally before entering base of oötype (Fig. 3A). Oötype ascending limb curved, looped or straight 240 ± 42.9(183–376, n = 25) long (following curvature) with valve opening into common space posterior to unarmed common genital pore (Fig. 3A). Small unarmed vaginal pore opens on left side of body in line with common genital pore, close to left branch of caecum (Fig. 3A). Unarmed, simple, unsclerotised vagina 200 ± 24.8(169–268, n = 17) long travels straight to roughly circular dorsoventrally bipartite seminal receptacle 67 ± 18.5(37–111, n = 25) in diameter (Fig. 3A). Spermatophores not observed. Vitellarium extends from level with posterior region of pharynx to posterior-most portion of body proper. Transverse vitelline duct thick (Fig. 3A).

DNA reference sequences: two identical ribosomal 28S DNA reference sequences (962 bp) representing *Scuticotyle cairae* **n. sp.** are deposited in GenBank under accession numbers OR351733 and OR351734. The result from the BlastN search (01.04.24) resulted in no identical or close hits. The p-distances (Supplementary file S2) show a difference of 60 nucleotides or greater between *S. cairae* **n. sp.** and all other monocotylid taxa. The ML-analyses clearly demonstrate the placement of *S. cairae* **n. sp.** as a new species representing a separate genus within the Troglocephalinae (Fig. 1).

Type-host: *Acroteriobatus annulatus* (Smith).

Type-locality: Muizenberg, False Bay, Cape Town, South Africa (34°06’13”S, 18°29’00”E).

Additional locality: Off Cape Agulhas, South Africa.

Microhabitat: Between secondary gill lamellae.

Etymology: This species is named in honour of Prof. Janine Caira of The University of Connecticut, Department of Ecology and Evolutionary Biology in recognition of her extensive contribution to helminthology.

Specimens: AHC 37031 (holotype); AHC 36032–37056 (25 paratypes); AHC 37057 (14 vouchers); AHC 37058 (3 digests).

ZooBank registration: urn:lsid:zoobank.org:act:09701C51-E75E-48C6-AC44-C8CAB1674877.

### Remarks

The proposal of *Scuticotyle* is supported by the 28S ML-analyses and the combination of morphological features of the body-proper, the sclerotised male copulatory organ, and the vagina. *Scuticotyle* differs from all representatives of Troglocephalinae by the presence of the posterior shield-like projection of the body-proper (Fig. 2A), and the unique morphology of the male copulatory organ, which is reduced to thinly sclerotised adjacent walls exiting the musculo-glandular ejaculatory bulb and is not surrounded by a muscular sheath. The thinly sclerotised nature of these walls was assessed by proteolytic digestion and is confirmed in the staining using Gomori’s Trichrome. The reduction of the male copulatory organ is peculiar; however, the presence of a field of papillae near the vaginal pore on the ventral tegument, suggests that this species likely produces external spermatophores, perhaps similar to those described for the microbothriid *Dermopristis cairae* Kearn, Whittington & Evans-Gowing, 2011, which are attached to the ventral tegument near the vaginal pore (Kearn et al. 2011). No spermatophores were observed in our specimens but spermatophores are known from this subfamily, from *Ne. rhynchobatis* (Chisholm and Whittington 1997). *Scuticotyle* is most similar to the genera *Mehracotyle*, *Troglocephalus*, and *Brancheocotyle* **n. gen.**, all of which have seven radial haptoral loculi, no dorsal haptoral accessory sclerites, and a bipartite seminal receptacle (see Young 1967; Neifar et al. 2002 for this detail in *Troglocephalus* and *Mehracotyle*, respectively). The sclerotised male copulatory organ of *Brancheocotyle* **n. gen.** is also void of a muscular sheath, but this feature is present in *Mehracotyle* and *Troglocephalus*. The sclerotised male copulatory organ length of *Scuticotyle*, *Brancheocotyle* **n. gen.** and *Mehracotyle* is short, and longer in *Troglocephalus*. The vagina of *Scuticotyle* is a simple, inconspicuous, narrow, non-musculo-glandular tube but is musculo-glandular in *Brancheocotyle* **n. gen.** and *Troglocephalus.* The vagina is apparently missing in *Mehracotyle* (Neifar et al. 2002). It is possible that the vagina is present in *Mehracotyle*, and if so, that it too is a simple, inconspicuous tube, because this species has a seminal receptacle that was confirmed by Neifar et al. (2002) to contain sperm. Neither *Scuticotyle* nor *Mehracotyle* have a septal ridge along any of the haptoral septa. Additionally, *Scuticotyle* has fewer anterior head region gland-duct openings (twelve or thirteen on either side) than *Mehracotyle* (twenty-six to thirty-three on either side; Neifar et al. 2002) and *Troglocephalus* (fifteen to twenty on either side) but more than *Brancheocotyle* **n. gen** (six or seven on either side). *Mehracotyle* is the only member of the subfamily with an ovary that does not loop the right intestinal caecum. The radial loculi are approximately of equal size in *Scuticotyle,* and *Brancheocotyle* **n. gen.** but the posterior haptoral loculus of *Mehracotyle* is notably much larger than any of its other haptoral loculi, and its posterolateral loculi are larger than the anterior and anterolateral loculi. *Troglocephalus* has dendritic structures within the marginal membrane of the haptor and an accessory piece associated with the hamulus, both absent in *S. cairae* and the other species.

***Brancheocotyle*** **n. gen.**

Generic diagnosis: Anterior region of body with three anterior glands; anteromedian gland and anterolateral glands. Numerous large, singular, pad-like marginal anterior gland-duct openings present. Eight anterior ventral pits present. Paired anterior dorsal pits present. Granulated eyespots present. Haptor roughly circular with one central and seven peripheral loculi. Septal ridge present. Haptoral papillae absent. Septal or dorsal haptoral accessory sclerites absent. Marginal membrane present. Fourteen marginal hooklets distributed in the marginal membrane. Hamuli robust with reduced superficial root and long, thick dorsoventrally compressed deep root with deep longitudinal grooves, without accessory piece. Testis ovoid, medial. Musculo-glandular ejaculatory bulb present. Sclerotised male copulatory organ present, without accessory piece, accessory filament, or muscular sheath. Ovary with radial lobes overlapping testis ventrally. Ovarian branch loops around right intestinal caecum. Caecum without diverticula; non-confluent posteriorly. Unarmed vagina with conspicuous, unsclerotised muscular inner walls and surrounded in radial glandular tissue. Oötype straight. Common genital pore unarmed. Gill parasites of Rhinobatidae.

Etymology: Named for the gill microhabitat, after the Latin *branchiae* for gills.

Type and only species: *Brancheocotyle imbricata* **n. sp.**

ZooBank registration: urn:lsid:zoobank.org:act:063EC412-E434-472A-BABE-4DCEDAC64760.

**Fig. 5:**
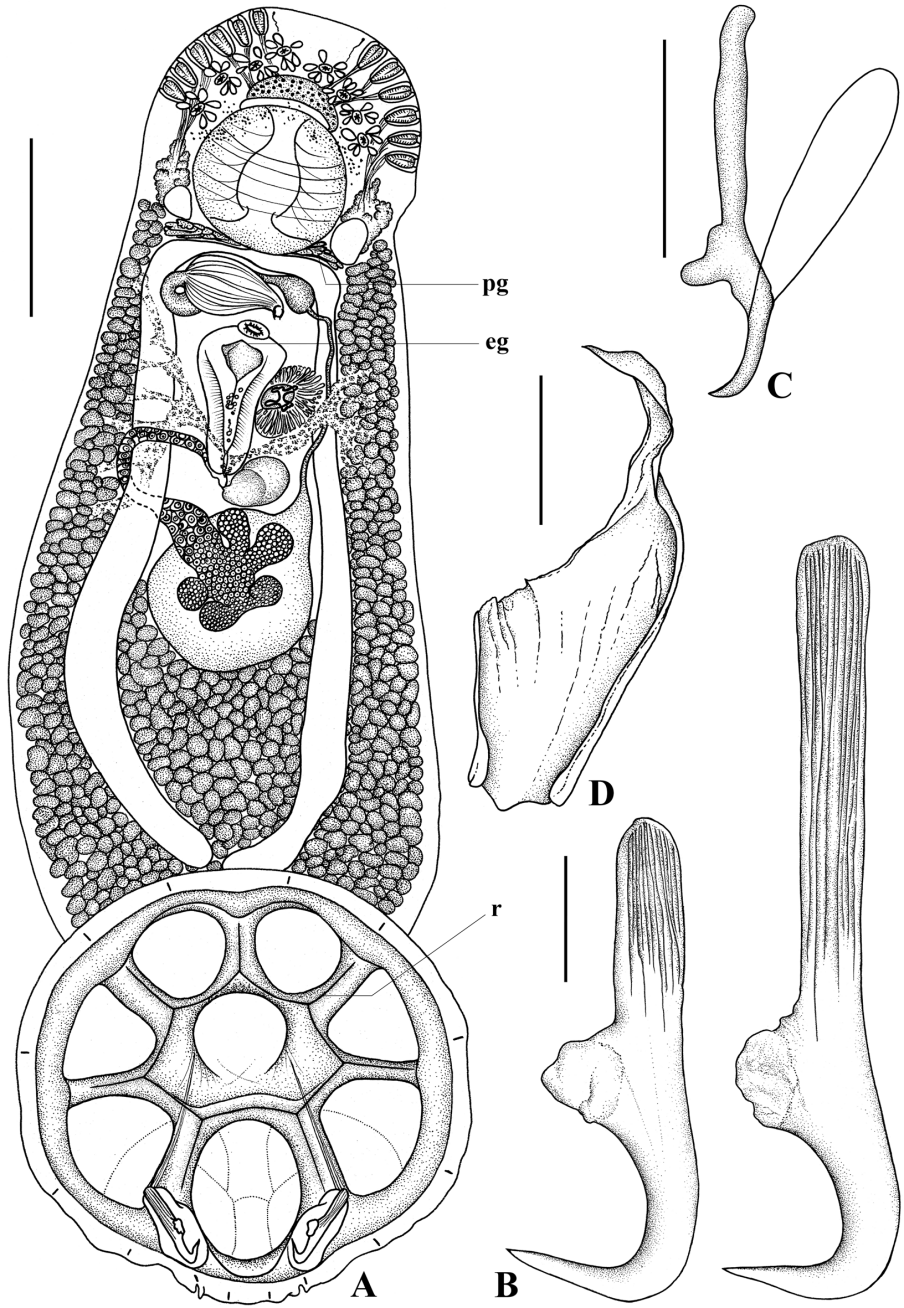
*Brancheocotyle imbricata* **n. gen. et sp.** A. Whole mount ventral view. B. Hamulus, demonstrating variation in length of handle in the size range of adult specimens observed. C. Marginal hooklet, D. Sclerotised male copulatory organ. Abbreviations: eg, egg; pg, pharyngeal glands; r, raised septal ridge. Scale bars: A = 300 µm, B = 100 µm, C, D = 10 µm.

**Fig. 6:**
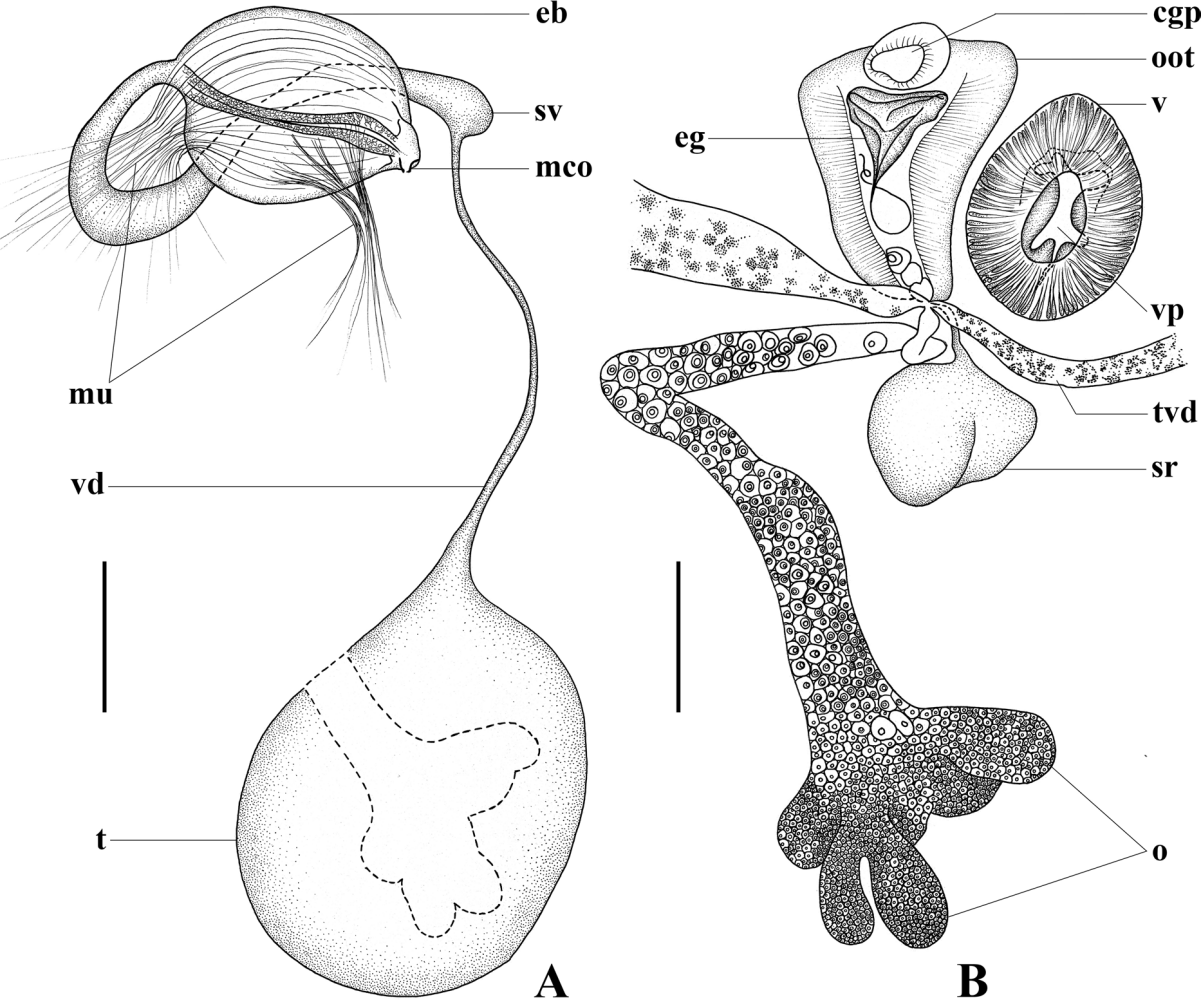
*Brancheocotyle imbricata* **n. gen. et sp.** Reproductive structures. A. Male reproductive complex. B. Female reproductive complex. Abbreviations as for Figs 1–4. Scale bars = 100 µm.

***Brancheocotyle imbricata* n. sp.** (Figs 5, 6)

Description. Based on the flat-preserved whole-mounted holotype and 11 paratypes, 3 digests and observations made from live specimens. Total body (excluding haptor) 1568 ± 356.5(1201–2500, n = 12) long, 644 ± 150.9(409–968, n = 12) wide at position of testis. Haptor roughly circular 716 ± 122.0(578–1020, n = 12) long, 748 ± 123.5(608–1042, n = 12) wide; divided into one central and seven peripheral loculi (Fig. 5A). Single, straight septal ridge present on inner-ring and radial septa, forming conspicuous heptagon around shallow central loculus (Fig. 5A). Marginal membrane present. Robust hamulus 198.4 ± 12.0(177–217, n = 12) long with reduced superficial root ending irregularly; deep root thick, notably anteroventrally compressed and deeply grooved; accessory piece absent (Fig. 5B). Deep root of hamulus length influenced by size of individual worms (Fig. 5B). Fourteen marginal hooklets with thick handle, 19 ± 0.4(18–19, n = 12) long with domus situated in the marginal membrane, as illustrated (Figs 5A, C). Mouth ventral, subterminal. Anterior head region with six or seven pairs of gland-duct openings with ventral pad-like termini situated marginally (Fig. 5A). Four pairs of ventral pits present, flanked dorsally by sets of smaller ovoid dorsal pits (Fig. 5A). Three anterior glands present (Fig. 5A), type of secretions not observed. Anteromedian gland positioned medially and immediately anterior to mouth (Fig. 5A). Two pairs of fine ducts lead laterally from anteromedian gland travelling between anterior ventral pits; anterior-most service anterior-most pair of marginal gland-duct openings; posterior pair of fine ducts splits into two to service next two consecutive pairs of marginal gland-duct openings (Fig. 5A). Remaining marginal gland-duct openings serviced by network of fine ducts originating from pair of anterolateral glands on either side of large ovoid pharynx (Fig. 5A). Pharyngeal glands present (Fig. 5A). pharynx muscular, 198 ± 51.8(155–350, n = 12) long, 218 ± 50.5(160–360, n = 12) wide (Fig. 5A). Oesophagus not observed. Intestinal caecum bifurcates immediately post-pharynx; excretory bladders adjacent to initial posteriorly directed curve (Fig. 5A). Both parallel caecal branches without diverticula, non-confluent, curving posteriorly towards each other, ending in posterior portion of body proper in line with anterior part of haptor (Fig. 5A). Eyespots in form of dispersed pigment granules on dorsal surface, lateral and over anterior region of pharynx (Fig. 5A). Large ovoid testis 279 ± 31.3(239–264, n = 12) long, 308 ± 47.9(239–399, n = 12) wide (Figs 5A, 6A). Weakly sinuous vas deferens originates from left anterior part of testis, narrows and travels anteriorly, left of vagina before swelling abruptly to form seminal vesicle (Figs 5A, 6A). Seminal vesicle extends dorsally over ejaculatory bulb, enters its base on right side of body (Figs 5A, 6A). Ejaculatory bulb musculo-glandular, 158 ± 41.1(97–216, n = 12) long, 114 ± 22.1(86–157, n = 12) wide. Sclerotised male copulatory organ short, 30 ± 4.7(22–38, n = 11) long, gutter-like with weak curvature, without accessory piece (Figs 5D, 6A). Lobate ovary situated directly ventral to testis with 3–5 (n = 12) radial lobes (Figs 5A, 6B). Single ovarian branch loops right intestinal caecum dorsoventrally, narrowing as it enters base of oötype (Figs 5A, 6B). Mehlis’ gland not observed. Oötype straight 256 ± 52.0(196–358, n = 12) long opens at unarmed common genital pore situated medially, immediately posterior to ejaculatory bulb and male copulatory organ (Figs 5A, 6B). Unarmed vaginal pore positioned immediately left of oötype (Figs 5A, 6B). Thick muscular folds of inner vaginal wall conspicuous immediately sub-vaginal pore (Fig. 6B). Vagina surrounded in radial, glandular tissue (Fig. 6B). Duct connecting proximal part of vagina and seminal receptacle not observed. Seminal receptacle weakly bipartite, ovoid 70 ± 24.9(41–138, n = 12) long, 118 ± 25.3(76–163, n = 12) wide. Ovovitelline duct short. Spermatophores not observed. Vitellarium extends from level of pharynx to posterior-most part of body proper. Transverse vitelline duct joins ovovitelline duct ventrally (Fig. 6B). Tetrahedral egg with single polar filament (Fig. 6B) observed in oötype of most specimens. Egg side 93 ± 4.3(87–101, n = 8) long, measured in the oötype.

DNA reference sequences: two identical ribosomal 28S DNA reference sequences (966 bp) representing *Brancheocotyle imbricata* **n. sp.** are deposited in GenBank under accession numbers OR351735 and OR351736. The result from the BlastN search (01.24.24) resulted in no identical or close hits. The p-distances (Supplementary file S2) show a difference of 70 nucleotides or greater between *B. imbricata* **n. sp.** and all other monocotylid taxa. The ML-analyses clearly demonstrate the placement of *B. imbricata* **n. sp.** as a new species representing a separate genus within the Troglocephalinae (Fig. 1).

Type-host, localities and microhabitat as for *S. cairae*.

Etymology: The species is named for the unique overlapping nature of the testis and ovary. The Latin word for overlapping is *imbricata*.

Specimens: AHC 37059 (holotype); AHC 37060–37070 (11 paratypes); AHC 37021 (6 vouchers); AHC 37072 (3 digests).

ZooBank registration: urn:lsid:zoobank.org:act:CC299745-AA48-4C75-9047-C59B889B6B0D.

### Remarks

The proposal of *Brancheocotyle* is supported by the ML-analyses of the two 28S sequences, and the combined morphology of the ovary that has radial lobes situated ventrally directly over the testis, the musculo-glandular morphology of the vagina, the morphology of the male copulatory organ, and the nature of the hamulus, which is reminiscent of the monotypic *Cathariotrema selachii*, having a reduced and irregularly truncated superficial root and a compressed, broad deep root with longitudinal grooves. Recently, *C. selachii* was redescribed by Bullard et al. (2021) demonstrating some morphological variability of isolates from different host species. *Cathariotrema selachii* is a member of Cathariotrematinae and differs markedly to *Brancheocotyle* in the general morphology of the anterior head region, the haptor, and has paired vaginal pores. Morphologically, the most similar genera are *Mehracotyle*, *Scuticotyle*, and *Troglocephalus*, all of which have seven peripheral haptoral loculi, no dorsal haptoral accessory structures, and a bipartite seminal receptacle. The hamuli of *Mehracotyle*, *Scuticotyle*, and *Troglocephalus* all have a narrow deep root, and the superficial root of *Mehracotyle* and *Scuticotyle* is well-developed. The superficial root of *Troglocephalus* is considered reduced (Chisholm et al. 1995) but it is not irregularly truncated. *Troglocephalus* also has a hamular accessory piece that is associated with the hook portion of the hamulus, absent in the other taxa. The ovary of *Brancheocotyle* is unique in the Monocotylidae in that it is proximally lobed and lies directly ventral over the testis. A lobed ovary is present in other monocotylids, notably *Peruanocotyle* species (Dasybatotreminae); however, the ovary of *Peruanocotyle* does not overlap with its four testes. *Mehracotyle* and *Scuticotyle* have a simple, unlobed ovary. *Troglocephalus* has an unlobed, weakly sinuous or irregular-shaped proximal portion of the ovary. The ovary of *Mehracotyle* does not loop the right intestinal caecum but it does in *Brancheocotyle*, *Scuticotyle*, and *Troglocephalus*. The vagina of *Brancheocotyle* is strongly muscular and is surrounded by conspicuous glandular tissue. The vagina of *Troglocephalus* is also musculo-glandular; however, the vagina is not musculo-glandular in *Scuticotyle* (see the remarks section for this taxon for the comparative discussion on the purported absence of a vagina in *Mehracotyle*). The sclerotised male copulatory organ of *Brancheocotyle*, *Scuticotyle* and *Mehracotyle* is short, and is surrounded by a muscular sheath in *Mehracotyle* and *Troglocephalus* only. Additionally, a single, straight haptoral septal ridge is present in *Brancheocotyle* and *Troglocephalus* (confirmed in the present study) but is absent in *Mehracotyle* and *Scuticotyle*. The latter genus includes an additional shield-like structure extending beyond the posterior portion of the body-proper, absent in all other taxa. The marginal hooklets of *Brancheocotyle* appear to be larger and more robust than those of the other taxa.

**Dasybatotreminae** Bychowsky, 1957.

Revised diagnosis. Anterior head region roughly circular in shape, with numerous, parallel radiating grooves or parallel radiating rows of multiple small gland-duct openings, present along the majority of the circumferential margin. Anterior-most part of head region with or without anterior notch. Eight anterior ventral pits absent. Eyespots present or absent. Pharynx muscular, ovoid. Intestinal caeca diverticular or non-diverticular, non-confluent posteriorly. Male copulatory system with musculo-glandular ejaculatory bulb with or without bipartite internal portion of seminal vesicle. sclerotised male copulatory organ, with or without accessory piece, without accessory filament. One or four ovoid testes present. Ovary positioned anterior to testis/testes simple or proximally lobed; distal branch highly convoluted, or not, looping right intestinal caecum. Vagina single; vaginal wall sclerotised or not; vaginal pore unarmed. Oötype with ascending limb only. Common genital pore armed or unarmed. Haptor roughly circular with one central and seven or eight peripheral loculi. Marginal membrane present. Hamuli present with short or elongated deep root; superficial root well-developed or reduced; accessory piece present or absent. Fourteen marginal hooklets distributed in the marginal membrane. Marginal haptoral papillae present or absent. Papillary sclerites, septal sclerites, dorsal haptoral protuberances or dorsal haptoral accessory sclerites absent. Single non-sinuous septal ridge present or absent. Parasites of the gills and pharyngeal cavity of myliobatiform stingrays and rajiform skates.

Type-genus: *Dasybatotrema* Price, 1938.

Other subordinate taxa (monotypic genus indicated by the species): *Timofeevia rajae* Chisholm, Wheeler & Beverley-Burton, 1995, *Peruanocotyle* Chero, Cruces, Sáez & Luque, 2018.

### Remarks

**Fig. 7:**
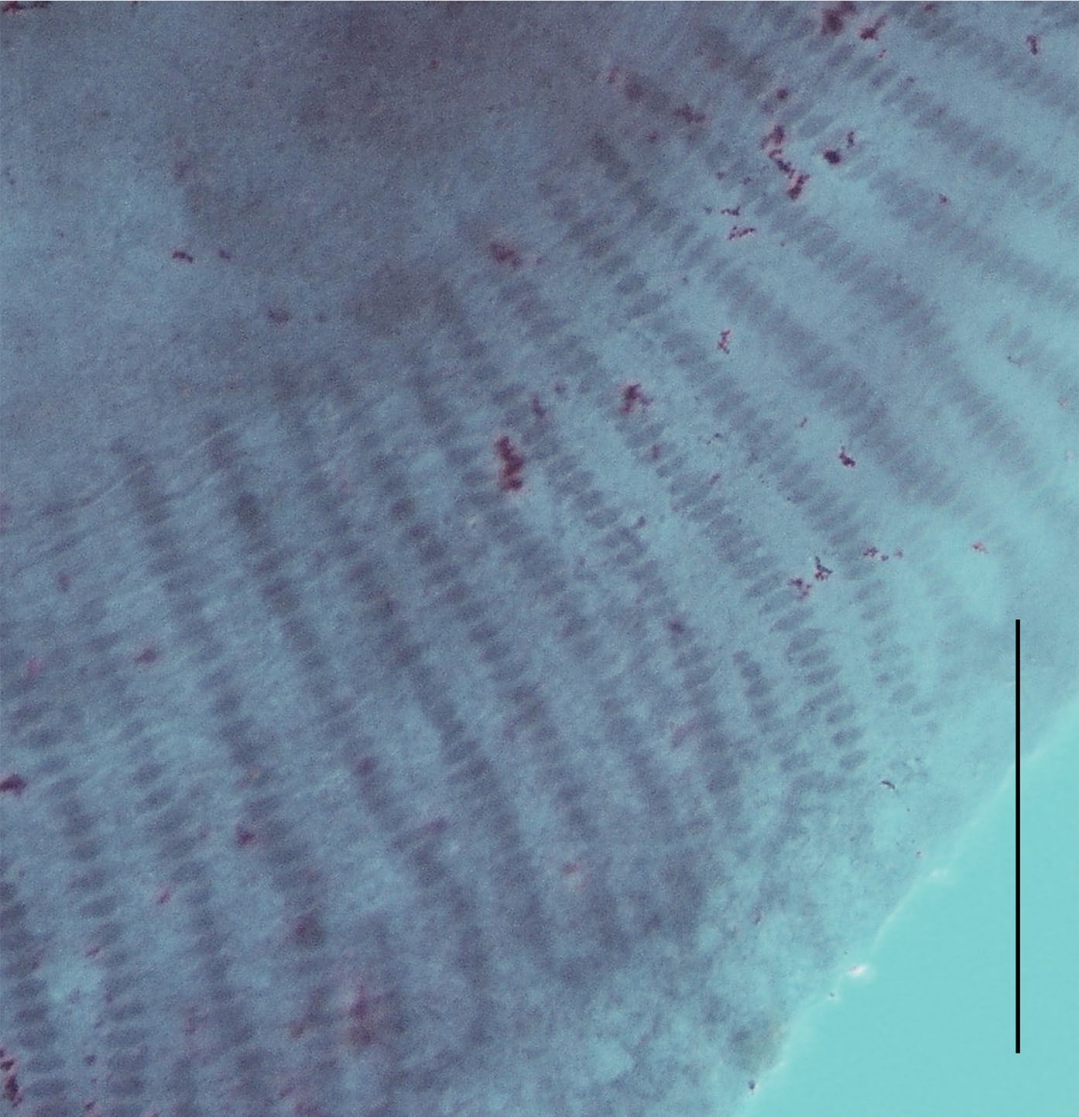
Portion of anterior head region of *Dasybatotrema dasybatis* voucher HWML 17719_17164, demonstrating the detail of the parallel rows of gland-duct openings radiating around the margin. Scale bar = 100 µm. Photomicrograph taken by Professor Marcus Vinicius Domingues.

The ‘short’ nature of the deep hamular root in Dasybatotreminae is represented only in *P. chisholmae*. It is elongate in all other members of the subfamily. Chisholm et al. (1995) included that the length of the hamulus deep root for the group, was greater than half that of the radial haptoral septa, and that the superficial root was either reduced or well-developed. These authors also included that the absence of a sinuous haptoral septal ridge, the absence of septal, papillary, and dorsal haptoral accessory structures (either sclerotised or not), and a non-diverticular ceacum, were typical in this group. *Peruanocotyle* species have very small hamuli, which prompted Chero et al. (2018) to amend the description of this character state to the deep root length being greater than half the length of the radial septa or shorter than the width of the marginal membrane. Chero et al. (2018) also included the presence of the diverticular caecum, and the presence of four testes, for *Peruanocotyle*. All representatives of Dasybatotreminae have the characteristic radiating arrangement of parallel rows of grooves, or gland-duct openings around nearly the entire circumference of the anterior head region (see representations in Price 1938 for *D. dasybatis*, Timofeeva 1983 for *D. spinosum* and *T. rajae*, Chero et al. 2018 for *P. chisholmae*, and Ruiz-Escobar et al. 2022 for *P. pelagica*). The morphology of these parallel radiating grooves or gland-duct openings is unique in this group. The stained voucher specimen HWML 17119_17164 clearly demonstrates that these parallel radiating rows in *D. dasybatis* are made up of many very small, separate individual gland-duct openings (Fig. 7). These differ significantly in form from the large, conspicuous, singular, marginal gland-duct openings of Troglocephalinae representatives. Although Chero et al. (2018) and Ruiz-Escobar et al. (2022) did not evaluate the glandular nature of these grooves in *Peruanocotyle* species, Chero et al. (2018) did detail the presence of three large head glands in *P. chisholmae*, which are associated with gland-duct openings in other representative monocotylid subfamilies. Given that there are no other structures present that would discount these radiating grooves or rows of gland-duct openings as glandular in nature, we consider them analogous to the gland-duct openings in other members of the family. Chero et al. (2018) included an unarmed common genital pore character, and the presence or absence of an accessory filament associated with the male copulatory organ in their revised subfamily diagnosis for Dasybatotreminae; however, the type-genus, *Dasybatotrema* contains species with an armed common genital pore (Chisholm et al. 1995), and the presence of an accessory filament was based on its erroneous inclusion for *A. papillatus* by Chisholm et al. (1995), now a representative of Troglocephalinae. A bipartite portion of the seminal vesicle, internal within the ejaculatory bulb, was described by Chero et al. (2018) for *P. chisholmae* and is also demonstrated by Timofeeva (1983) for *T. rajae*.

## Discussion

The proposal of Troglocephalinae was justified in the current study because the two new *Troglocephalus*-like species share common morphological features with related taxa that were previously, and problematically included within Dasybatotreminae and Heterocotylinae. The representative subfamily group in our phylogeny presents *Neoheterocotyle* as paraphyletic (Fig. 1). We have specifically labelled AF026107 as ‘*Neoheterocotyle* sp.’ in the phylogeny, because to the best of our knowledge, Mollaret et al. (1997) did not provide a confirmatory morphological anchor for ‘*Ne. rhinobatidis* AF026107’ in the form of a definitive identity, or the deposition of any verifiable representative museum specimens for their material. Multiple *Neoheterocotyle* species are known to infect the same host species and host individuals (see Chisholm and Whittington 1997; Kritsky and Chisholm 2020), including *Glaucostegus typus* from which AF026107 originated in the study by Mollaret et al. (1997). This, and the fact that Nitta (2019) recently found only a 92.7% similarity between the two available ‘*N. rhinobatidis*’ sequences, suggests that AF026107 represents a separate *Neoheterocotyle* species. Consequently, we consider the sequence AF026107 nonugen as per Roberts et al. (2018) and treat it conservatively as an unnamed species of *Neoheterocotyle* in the phylogeny. This sequence was excluded from the phylogenetic analyses of Chisholm et al. (2001a), Fehlauer-Ale and Littlewood (2011), Vaughan et al. (2016), Derouiche et al. (2019), Chero et al. (2021), and Bullard et al. (2021). An additional problematic sequence which has been used repeatedly in historic phylogenies to represent *Neoheterocotyle*, is AF348362, representing *N. rhinobatis*, originating from Chisholm et al. (2001a). The late A/Prof. Ian Whittington considered this sequence to be erroneous (pers. comm.). We investigated the historic, meticulous field notes and data originating from the study of Chisholm et al. (2001a). Of all the taxa listed in Chisholm et al. (2001a), this species was the only one for which we could not find any confirmatory morphological identity data, or data verifying the identity or collection date of the purported host species. It is clear from the current phylogeny that AF348362 represents a member of the Monocotylidae, but that it is not a *Neoheterocotyle* species (Fig. 1). The p-distance matrix (Supplementary file S2) indicates 97–119 nucleotide differences between AF348362 and all other *Neoheterocotyle* sequences. The two new species represent an apparent sister group to the group containing *Ne. rhinobatidis*, *Ne. rhynchobatis*, *Ne. robii*, *Ne*. sp., *Ne. quadrispinata*, and *Tr. rhinobatidis*, however at less than 95% bootstrap support (Fig. 1). These sister taxa form a monophyletic group separate from the monocotylid represented by AF348362 (Fig. 1). We consider the sequence AF348362 to represent Monocotylidae sp. *incertae sedis* and have amended the accession details for AF348362 in GenBank to represent Monocotylidae sp. *Neoheterocotyle* requires careful future revision. The Troglocephalinae is supported in the current phylogeny with ventral pits of the anterior head region as an apomorphy for the group. The net evolutionary divergence between Troglocephalinae and the other subfamilies is represented in Table 2. Troglocephalinae is less divergent from Monocotylinae (52.8 nucleotide bases) and Decacotylinae (49.6 nucleotide bases) than Dasybatotreminae (100.6 nucleotide bases) and Heterocotylinae (77.5 nucleotide bases). Heterocotylinae is more divergent from Troglocephalinae than Monocotylinae (57.4 nucleotide bases). Troglocephalinae is most divergent from Calicotylinae (113.5 nucleotide bases) and Cathariotrematinae (113.9 nucleotide bases) and is approximately similar in divergence from Dasybatotreminae as it is from Merizocotylinae (102.6 nucleotide bases), which is similar between Decacotylinae and Dasybatotreminae (98.7 nucleotide bases). The evolutionary divergence of 100.6 nucleotide bases between Troglocephalinae and Dasybatotreminae, and 77.5 between Troglocephalinae and Heterocotylinae further support the justification of the proposal of the new subfamily and the amendment of Dasybatotreminae and Heterocotylinae.

**Table 2:** Estimates of net evolutionary divergence between sequences for subfamilies, Monocotylidae sp. *incertae sedis*, and the outgroup

	Calicotylinae	Cathariotrematinae	Dasybatotreminae	Decacotylinae	Heterocotylinae	Loimoinae	Merizocotylinae	Monocotylinae	Troglocephalinae	Monocotylidae sp.
Cathariotrematinae	108.22									
Dasybatotreminae	167.69	151.58								
Decacotylinae	100.43	108.75	98.67							
Heterocotylinae	108.98	121.08	123.40	60.77						
Loimoinae	128.81	136.04	128.17	63.83	82.99					
Merizocotylinae	76.36	76.93	152.78	92.26	97.45	121.15				
Monocotylinae	92.71	85.95	104.48	45.33	57.41	68.33	75.99			
Troglocephalinae	113.53	113.86	100.61	49.61	77.52	70.54	102.59	52.79		
Monocotylidae sp.	128.05	133.08	123.00	77.00	92.75	96.67	126.42	78.05	67.28	
Outgroup	161.81	170.83	172.67	142.00	155.30	153.89	141.47	105.10	138.28	163.67

The number of base differences per sequence from estimation of net average between groups of sequences are shown. The rate variation among sites was modelled with a gamma distribution (shape parameter = 1). This analysis involved 79 nucleotide sequences. All ambiguous positions were removed for each sequence pair (pairwise deletion option). There were a total of 1236 positions in the final dataset. Evolutionary analyses were conducted in MEGA11 (Tamura et al. 2021).

The transferral of *Neoheterocotyle* to the new subfamily addresses the historic polyphyly of the Heterocotylinae. The representatives of Heterocotylinae, as amended, reduces the known host association of the group to gill parasites of myliobatiform and torpediniform rays. *Heterocotyle* is however paraphyletic within Heterocotylinae with the inclusion of the new sequence for *Het. tokoloshei* (Fig. 1). *Heterocotyle capricornensis* is currently represented as a separate taxon to the rest of the heterocotylinids within the subfamily (Fig. 1); however, morphologically, this species is still considered a well-supported representative of the subfamily. This species was admittedly problematic in both morphological and molecular phylogenies presented by Chisholm and Whittington (1996) and Chisholm et al. (2001a). In the morphological phylogeny of the genus (Chisholm and Whittington 1996), *Het. capricornensis* was either placed in an unresolved trichotomy with all other *Heterocotyle* species, or grouped with the outgroups (*Ne. rhinobatidis*, *Nonacotyle pristis*, *Potamotrygonocotyle tsalickisi* Mayes, Brooks & Thorson, 1981, and *Spinuris lophosoma* Doran, 1953). This resulted from the unique combination of two characters states in *Het. capricornensis*: a single sinuous haptoral ridge, and a male copulatory organ without an accessory piece (Chisholm and Whittington 1996). In the molecular phylogeny of Chisholm et al. (2001a), *Het. capricornensis* was presented as a separate taxon, sister to Decacotylinae, or in a polychotomy between a group represented by *Troglocephalus* and three *Neoheterocotyle* species, and a group representing Decacotylinae. Chisholm et al. (2001a) urged the inclusion of additional *Heterocotyle* species into future molecular phylogenies to evaluate the validity of *Heterocotyle*. The inclusion of *Het. tokoloshei* provides only limited additional resolution, thus we cannot comment on the validity of the genus; however, Chisholm and Whittington (1996) did describe additional unique characters for *Het. capricornensis*: a tri-lobed testis, and a distal ovarian loop, anterior to the initial caecal ovarian loop. Whether these unique characters reflect a generic difference is debatable, although the current phylogenetic position of the two *Heterocotyle* species might support this hypothesis. Decacotylinae was represented as a sister group to Calicotylinae, Cathariotrematinae, and Merizocotylinae in Bullard et al. (2021); however, in the current molecular phylogeny, Decacotylinae is unresolved, representing *Decacotyle* as paraphyletic and as basal to all other members of the family (Fig. 1).

The Loimoinae was formally incorporated into the Monocotylidae by Chero et al. (2021) with the inclusion of *Loimopapillosum pascuali* Chero, Cruces, Sáez, Oliveira, Santos & Luque, 2021. In the earlier molecular phylogeny of Boeger et al. (2014), who opted to provisionally retain Loimoidae Price, 1936, this taxon, represented by *Loimosina* Manter, 1944 sp. (= *Loimosina wilsoni* Manter, 1944; see Dalrymple et al. 2022) was considered a sister group to *Tr. rhinobatidis* (previously as *incertae sedis*) and *Ne. rhinobatis* (Monocotylidae sp. *incertae sedis*; for the previously polyphyletic Heterocotylinae). Moreover, Boeger et al. (2014) considered the ventral pits of the anterior head region, which are present in *Loimos* MacCallum, 1917 and *Loimosina* as a synapomorphy for a group representing Loimoidae (*Loimosina wilsoni*), *Tr. rhinobatidis* and *Ne. rhinobatis* (Monocotylidae sp. *incertae sedis*). In our phylogenetic analysis, the presence of ventral pits as a synapomorphy is also the most parsimonious hypothesis, where this character is secondarily lost in Dasybatotreminae (represented by *Peruanocotyle*), but also in *Loimopapillosum* Hargis, 1955. Boeger et al. (2014) also suggested a possible phylogenetic relationship between *Me. insolita* (for Dasybatotreminae) and Loimoidae (*Loimosina wilsoni*) based on the lack of the ovarian branch looping the right intestinal caecum. Chero et al. (2021) also recognised the possible phylogenetic relationship between *Loimos*, *Loimosina*, *Me. insolita* (for Dasybatotreminae) and *A. papillatus* (for Dasybatotreminae), based on the ventral pits and the non-looping ovarian branch. However, their inclusion of *Loimopapillosum pascuali* represented Loimoinae as they defined it, as paraphyletic, with *Loimopapillosum pascuali* as a sister group to *Het. capricornensis* (Heterocotylinae). Dalrymple et al. (2022) came to the same conclusion regarding the paraphyly of the loimoids, opting to take a more conservative approach to the group, refraining from classifying them as Loimoidae or Loimoinae until additional sequences could be included of representative members. The sequence for *Loimos* sp. is the first representative sequence for this genus to be included in a molecular phylogeny of the family (Fig. 1), and it groups together with *Loimosina* with high support. *Loimopapillosum* was reported previously as a representative of the Loimoinae (Chero et al. 2021) but this was not supported in their presented phylogenies based on 28S rDNA sequences. *Loimopapillosum* grouped together with *Loimosina* in the 18S phylogeny of Chero et al. (2021); however, this phylogeny was based on a very restricted number of taxa. Dalrymple et al. (2022) included *Loimopapillosum* in a larger phylogeny, also based on 28S, and clearly demonstrated that *Loimopapillosum* and *Loimosina* were not part of the same group. Similarly, *Loimopapillosum* is not supported in the current phylogeny as a representative of Loimoinae. We agree with Boeger et al. (2014) and Dalrymple et al. (2022) that additional work is required for this group before a decision can be made on assigning members of the group to only a single subfamily. *Loimopapillosum* is morphologically very different from *Loimos* and *Loimosina*, and there are at least 161–169 pairwise nucleotide differences between *Loimopapillosum pascuali* and *Loimosina wilsoni*, and 161 between *Loimopapillosum pascuali* and *Loimos* sp.; there are 64–73 nucleotide differences between *Loimosina wilsoni* and *Loimos* sp. (see Supplementary file S2). Based on the morphological differences between *Loimopapillosum* species and the other members of the group, and the clear separation of *Loimopapillosum* in the current phylogeny, *Loimopapillosum pascuali* is considered *incertae sedis*. Additional future data from new specimens of *Loimopapillosum* might justify the consideration of a separate subfamily to accommodate it.

Monocotylinae is not supported as a monophyletic group in the current phylogeny (Fig. 1). Similarly, the phylogenetic analysis of Bullard et al. (2021) and Dalrymple et al. (2022) presented low support for the Monocotylinae. The separation of the group containing *Dendromonocotyle* Hargis, 1955 and *Clemacotyle* Young, 1967 from the group containing *Monocotyle* Taschenberg, 1878 provides support for reinstating Dendromonocotylinae Hargis, 1955. This separation reflects the host microhabitats, where *Monocotyle* species are parasites of the gill tissue, and *Dendromonocotyle* and *Clemacotyle* are parasites of the external skin surface and gill cavity, respectively. Chisholm et al. (1995) were aware of the morphological support for Dendromonocotylinae; however, its formal recognition would have reduced Monocotylinae to the single genus based on homoplasy (see also Chisholm et al. 2001a); therefore, these authors chose to assign *Dendromonocotyle* and *Clemacotyle* to Monocotylinae. This decision was supported in the molecular phylogeny of Chisholm et al. (2001a), representing a monophyletic Monocotylinae. The relationship between *Dendromonocotyle* and *Clemacotyle* is still unresolved, and additional sequences but also a close re-evaluation of morphological characters within this subfamily are needed before an alternative treatment is proposed for Monocotylinae.

The current study includes a significant contribution to the known diversity of the Monocotylidae off South Africa and the phylogenetic resolution within the family. The inclusion of *B. imbricata* and *S. cairae*, *C. selachii*, *Het. pastinacae*, *Hel. kartasi*, and *My. pteromylaei*, doubles the previous number of described monocotylid species (Vaughan et al. 2021) to twelve, and increases the number of representative genera from this region from four to nine.

### Electronic supplementary material

Below is the link to the electronic supplementary material.

Supplementary file1 (FASTA 99 kb)

Supplementary file2 (XLSX 48 kb)

## Data Availability

All data used in this study are included in the article or associated supplementary files.
